# Response Surface Methodology for Biochar Performance Optimization: A Comprehensive Bibliometric Review

**DOI:** 10.3390/molecules31173055

**Published:** 2026-08-31

**Authors:** Chang Liu, Jian Wang, Ziyan Zhou, Mingqing Liu, Wu Lei, Fenghe Wang, Shengtian Zhang, Jing Hua, Jiaqi Shi

**Affiliations:** 1 Nanjing Institute of Environmental Sciences, Ministry of Ecology and Environment of China, Nanjing 210042, China; liuchang@nies.org (C.L.);; 2 School of Chemistry and Chemical Engineering, Nanjing University of Science and Technology, Nanjing 210094, China

**Keywords:** response surface methodology, biochar, preparation process, modification, adsorption performance, parameter optimization

## Abstract

Biochar, as a widely available and environmentally friendly porous carbonaceous material, holds broad application prospects in fields such as environmental remediation, soil improvement, and carbon sequestration. Its performance is highly dependent on the precise control of preparation processes and modification conditions. Traditional single-factor experiments and orthogonal designs cannot systematically reveal the interaction effects among multiple factors; they suffer from limitations such as a large number of experiments, low optimization efficiency, and a tendency to overlook global optimal solutions. Response surface methodology (RSM), which integrates experimental design, mathematical modeling, and parameter optimization, is an effective tool for solving complex optimization problems involving multiple factors and levels. Based on 1468 valid publications screened from the Web of Science Core Collection (2010–2025), this paper systematically reviews the development trajectory and research landscape of this field using bibliometric methods. The results show that the annual number of publications in this field increased from 4 in 2010 to 312 in 2025, with a compound annual growth rate of 33.7%. Research hotspots were concentrated in three major areas: optimization of adsorption performance, control of pyrolysis preparation processes, and chemical modification. Central composite designs and Box–Behnken designs were the most prevalent experimental approaches, accounting for 56.8% and 35.5%, respectively. Building on this foundation, this paper elucidates the basic principles of RSM and the applicability limits of commonly used design methods. It examines the optimization effectiveness and the value of analyzing interaction effects of RSM from three dimensions—biochar adsorption performance, preparation processes, and modification processes—in conjunction with typical application scenarios such as heavy metals, organic compounds, and non-metallic inorganic compounds; it also provides an in-depth analysis of core issues in current research, including limitations in secondary model fitting, inadequacies in multi-objective optimization, and significant extrapolation risks. Finally, the paper outlines future research directions, aiming to provide a systematic theoretical framework for the precise R&D and engineering applications of biochar materials.

## 1. Introduction

Biochar is a stable, porous carbonaceous material produced by the pyrolysis of biomass under oxygen-limited or anaerobic conditions. It is characterized by a large specific surface area, a well-developed pore structure, abundant surface functional groups, and high chemical stability [1]. In recent years, as global environmental pollution has become increasingly severe and the demand for carbon emission reduction has continued to rise, biochar has emerged as a research hotspot in fields such as environmental science, materials science, and agricultural science due to its advantages of widely available raw materials, low production costs, and environmental friendliness [2]. The performance of biochar directly determines its effectiveness in various applications. For example, when used for water pollution remediation, the specific surface area, pore volume, and number of surface oxygen-containing functional groups directly influence its adsorption capacity for heavy metal ions and organic pollutants [3]; when used for soil improvement, the pH, cation-exchange capacity, and nutrient contents of biochar determine its effectiveness in improving the physicochemical properties of soil [4].

The performance of biochar is influenced by a combination of factors, including preparation parameters such as feedstock type, pyrolysis temperature, pyrolysis time, and heating rate, as well as modification parameters such as activator type, activator concentration, activation temperature, and activation time [5]. The traditional single-factor experimental method examines the effect of a single factor on the response value by keeping other factors constant. Although simple to operate, this method fails to reveal the interactions among factors; moreover, when the number of factors is large, the number of experiments increases exponentially, leading to low optimization efficiency and potentially overlooking the optimal process conditions [6]. Although orthogonal experiments can reduce the number of trials, they only provide discrete optimization results and cannot yield a continuous response surface, making it difficult to accurately predict the optimal value [7].

Response surface methodology (RSM), first proposed by Box and Wilson in 1951, is an optimization method that combines statistics and mathematics [8]. It collects data through a well-designed experimental setup, uses multiple quadratic regression equations to fit the functional relationship between factors and response values, and identifies optimal process parameters by analyzing the regression equations, thereby effectively solving complex optimization problems involving multiple factors and levels. Compared with traditional experimental methods, RSM offers advantages such as fewer experiments, high accuracy, and the capacity to build predictive models. Furthermore, it can intuitively illustrate the effects of individual factors and their interactions on the response value through three-dimensional response surface plots and two-dimensional contour plots [6].

In recent years, the application of RSM in biochar performance optimization has become increasingly widespread, with a steady stream of related research findings [9,10]. However, most existing reviews either address biochar preparation and application in general or treat RSM as one technique among many rather than as the central object of analysis; in particular, Ref. [11] reviews modeling, design of experiments, RSM, and artificial intelligence for biochar process optimization, but it does not isolate RSM as the primary lens or systematically separate model fit from predictive performance. Few reviews specifically address the application of RSM in biochar performance optimization. To address this gap, this paper has three specific objectives: (1) to conduct a reproducible bibliometric analysis that maps the development trajectory, research hotspots, and collaborative networks of RSM-based biochar optimization research; (2) to systematically elaborate the basic principles of RSM and the applicability and limitations of commonly used experimental designs—including not only the widely used central composite design (CCD) and Box–Behnken design (BBD) but also alternative designs such as Doehlert, D-optimal/optimal, and sequential screening–optimization strategies; and (3) to critically synthesize, through representative case studies, how RSM has been applied to the optimization of biochar adsorption, preparation, and modification, while keeping the model fit, predictive performance, and mechanistic evidence distinct. Compared with prior reviews, this work generates new knowledge by explicitly distinguishing goodness of fit from predictive accuracy, identifying common failure modes of RSM applications, and proposing an integrated framework that couples RSM with machine learning.

## 2. Bibliometric Analysis

### 2.1. Data Sources and Search Strategy

This study used the Web of Science Core Collection (WoSCC) as the source database. The search was performed on 15 January 2026 in a single run across the following citation indices: Science Citation Index Expanded (SCI-EXPANDED), Social Sciences Citation Index (SSCI), Emerging Sources Citation Index (ESCI), and Conference Proceedings Citation Index—Science (CPCI-S). The search time range was set from 1 January 2010 to 31 December 2025. The search query was as follows: TS = (“response surface” OR “RSM”) AND TS = (“biochar” OR “biocarbon”) AND TS = (“optimization” OR “preparation” OR “modification”). The literature types were restricted to journal articles and review articles, and the language was restricted to English; non-academic literature such as conference papers, patents, and news reports was excluded.

### 2.2. Literature Analysis Methods and Screening Criteria

This study used Origin 2024 to analyze the annual publication volume of the literature, VOSviewer 1.6 for keyword co-occurrence analysis, and CiteSpace 7.0 for keyword clustering and emergence analysis, as well as author and institutional collaboration analysis.

**Figure 1:**
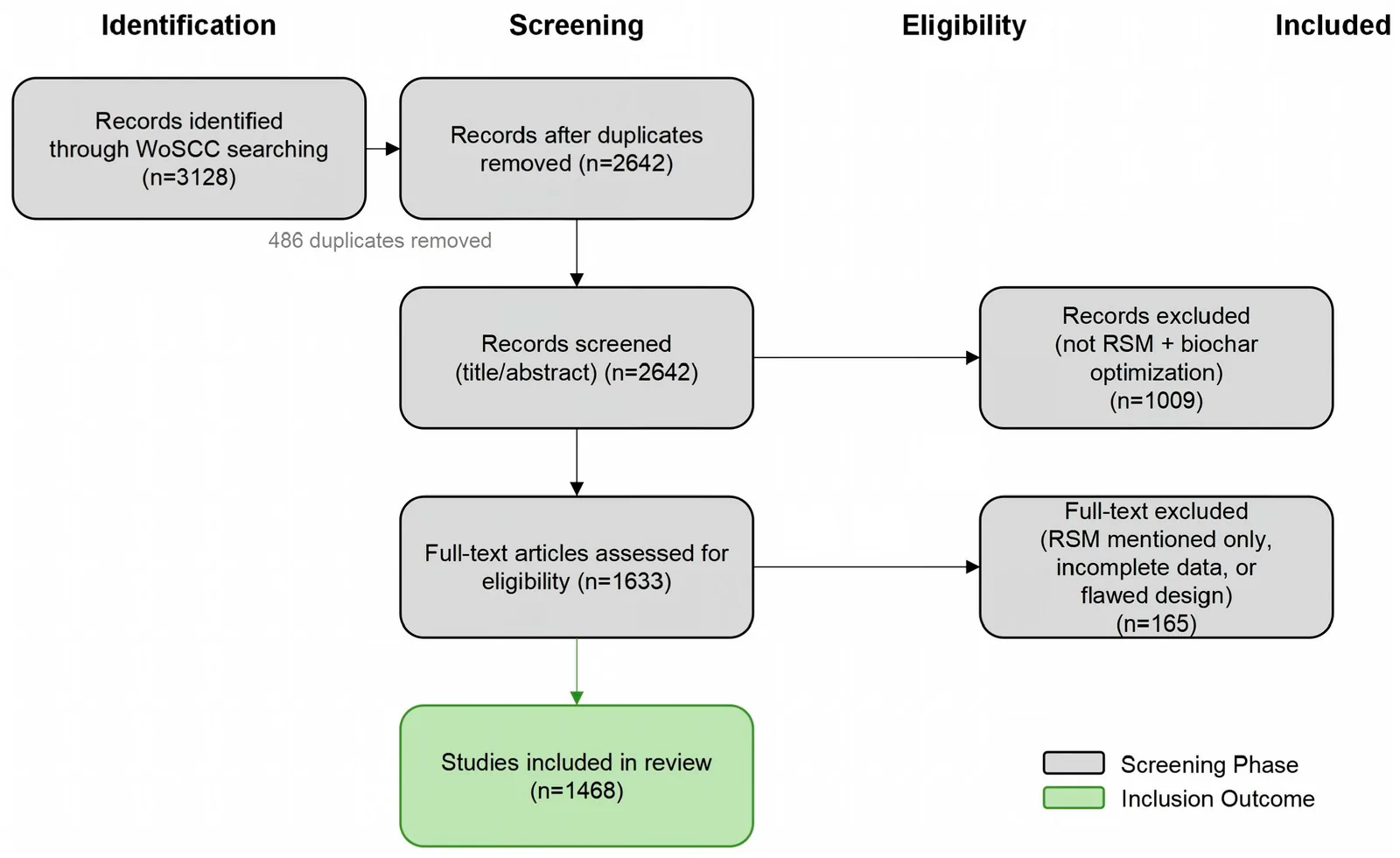
PRISMA flow diagram of the literature screening process.

To ensure the relevance and quality of the literature, the retrieved records were screened following the PRISMA (Preferred Reporting Items for Systematic Reviews and Meta-Analyses) framework (Figure 1). The initial search retrieved 3128 records, of which 486 duplicates were removed, leaving 2642 unique records. After screening titles and abstracts against the inclusion criteria, 1009 records were excluded because they did not involve both RSM and biochar performance optimization. The remaining 1633 full-text articles were assessed for eligibility, and 165 were further excluded because they merely mentioned RSM without actual application, or because they had incomplete data or obvious flaws in experimental design. A total of 1468 valid publications were ultimately selected for analysis.

### 2.3. Analysis of Bibliometric Results

#### 2.3.1. Analysis of Annual Publication Volume

**Figure 2:**
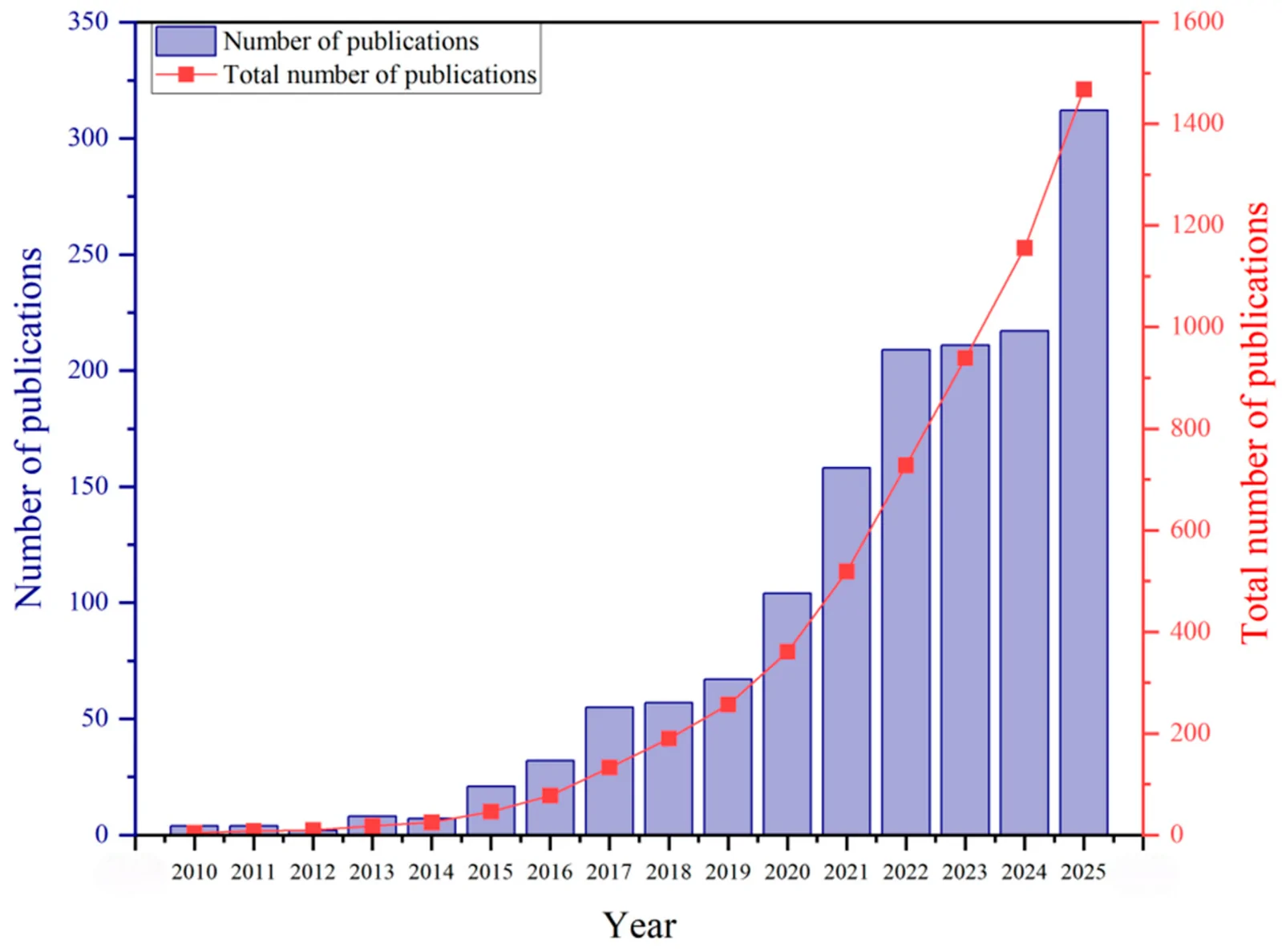
Annual trends in the number of publications on RSM in the field of biochar performance optimization, 2010–2025.

Figure 2 shows the trend in the annual number of RSM publications in the field of biochar performance optimization from 2010 to 2025. As shown in the figure, the annual number of publications in this field has generally shown a rapid growth trend, increasing from 4 papers in 2010 to 312 papers in 2025—a total increase of 78-fold, corresponding to a compound annual growth rate (CAGR) of 33.7%. This trend can be divided into three phases: (1) Initial Phase (2010–2014): During this phase, the number of publications was low, with an average of fewer than 10 papers per year. This was primarily because the application of RSM in the field of biochar was just beginning, and related research was still in its exploratory stage. (2) Rapid Growth Phase (2015–2021): As biochar research deepened and RSM became increasingly widespread, the number of publications grew rapidly, with an average annual growth rate of 42.1%. (3) Steady Growth Phase (2022–2024): The number of publications continued to rise, but the growth rate slowed somewhat, indicating that research in this field is gradually maturing, with studies becoming more in-depth and specialized. It is worth noting that the number of publications saw another significant increase in 2025 (up 43.8% compared to 2024).

#### 2.3.2. Keyword Co-Occurrence Analysis

**Figure 3:**
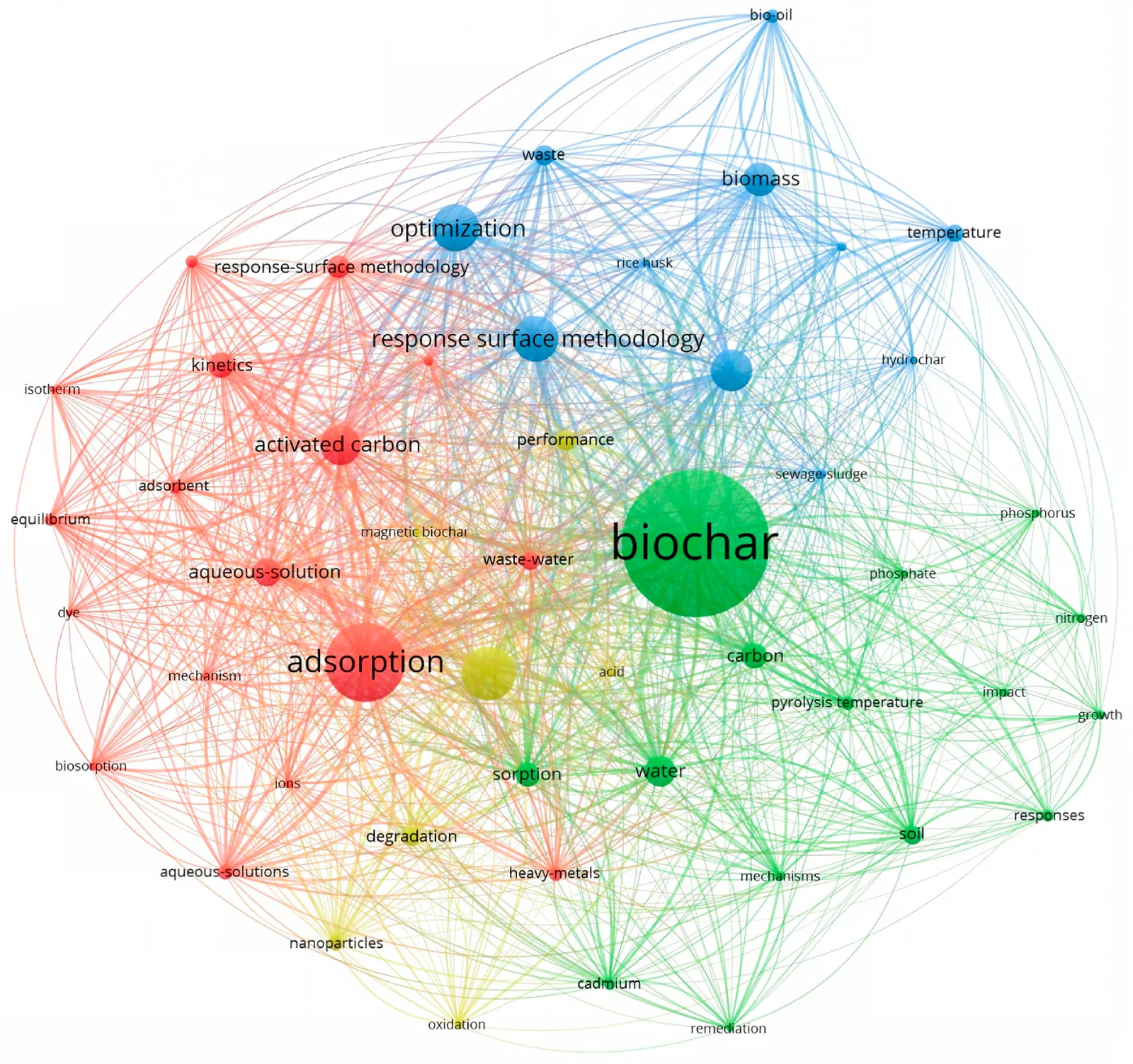
Keyword co-occurrence network diagram.

Keywords provide a concise summary of the literature’s core content; keyword analysis can reveal research hotspots in a given field. Figure 3 shows a keyword co-occurrence network diagram based on the WoSCC database. It presents 60 major keywords extracted from a total of 524 keywords; node size represents the frequency of keyword occurrence, and the lines represent the co-occurrence relationships between keywords. As shown in Figure 3, keywords with high occurrence frequencies include “biochar,” “adsorption,” “response surface methodology,” “removal,” “water,” “optimization,” and “pyrolysis.”

**Figure 4:**
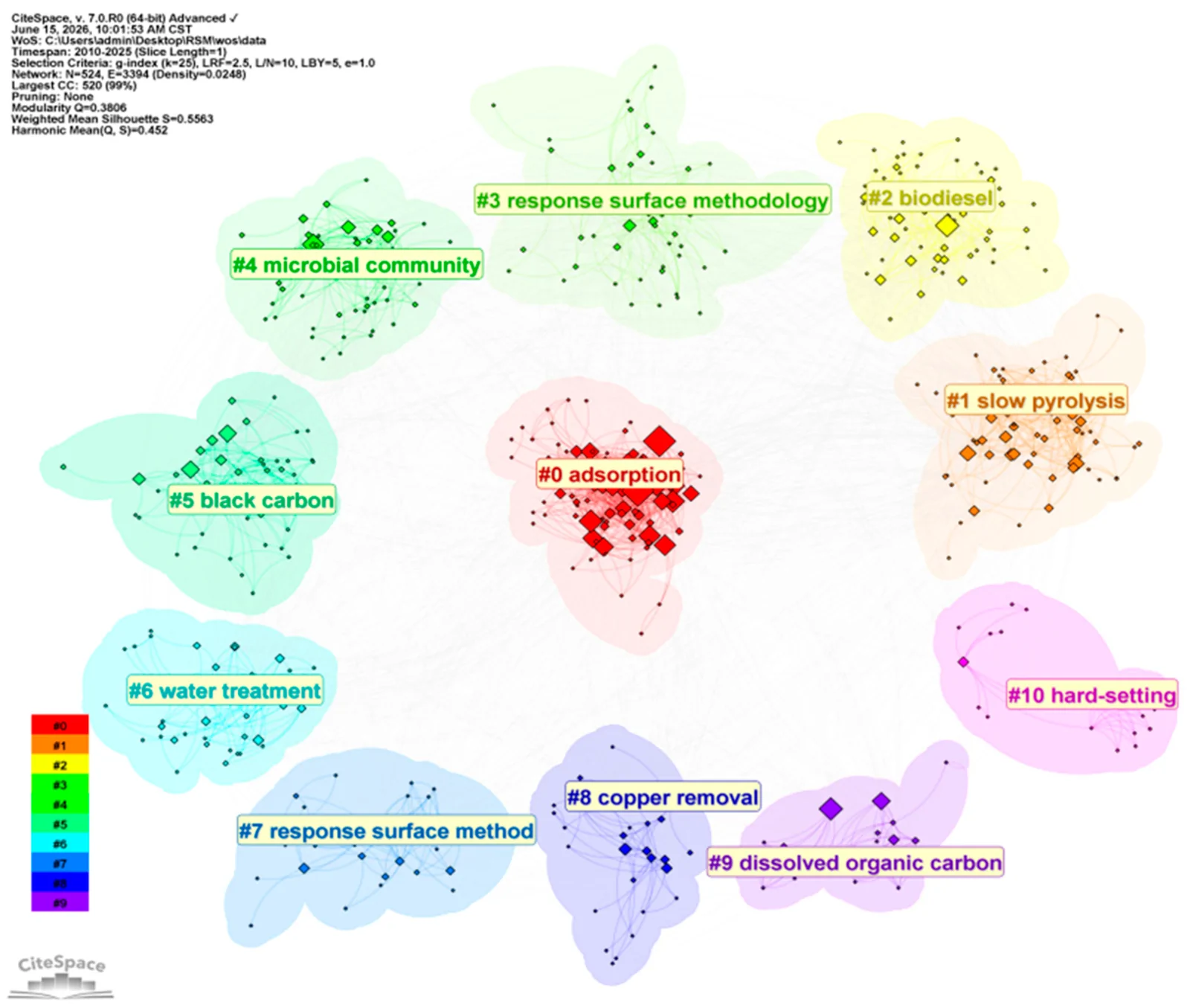
Keyword clustering map.

Based on a comprehensive analysis of the keyword clustering results (Figure 4, Table 1) and keyword co-occurrence frequencies, research hotspots in this field primarily focus on the following three areas: (1) The application of RSM in optimizing the adsorption performance of biochar, primarily investigating the optimization of biochar’s adsorption performance for heavy metal ions (e.g., Cu, Pb), organic pollutants (e.g., methylene blue), and non-metallic inorganic compounds (e.g., phosphates). (2) The application of RSM in optimizing biochar preparation processes, primarily investigating the effects of parameters such as pyrolysis temperature, pyrolysis time, and heating rate on biochar yield, specific surface area, and pore structure. (3) The application of RSM in optimizing biochar modification processes, primarily investigating the effectiveness of chemical activation, physical activation, and composite modification methods in enhancing biochar performance. The application scenarios for performance optimization are primarily concentrated in two major areas: the adsorption and treatment of water pollutants, and soil improvement and the enhancement of agricultural ecological quality and efficiency. At the same time, these applications extend to related fields such as carbon sequestration, ecological restoration, high-value utilization of biomass, and the catalytic production of bioenergy.

Keyword emergence analysis can systematically reveal the cutting-edge developments and hot-topic shifts in the field of RSM-optimized biochar performance research (Table 2, The red segments represent the citation-burst periods of keywords, while the teal segments indicate non-burst periods with relatively stable research popularity). In terms of emergence intensity and time series, the initial phase (2010–2014) centered on “charcoal” and “black carbon,” with emergence intensities as high as 13.26 and 9.40, respectively, reflecting the foundational status of basic biochar concepts. At this time, research in the field was still in its preliminary exploratory stage, with the academic community primarily focusing on the fundamental physicochemical properties of biochar [12]. Subsequently, the emergence of keywords related to preparation processes—such as “pyrolysis temperature” and “slow pyrolysis”—indicated a shift in the research focus toward the optimization of process parameters, marking the transition into a phase of rapid growth (2015–2021). As research in the field continued to evolve, terms such as “amendment,” “heavy metals,” and “enzyme activity,” signaling a shift from preparation processes to practical environmental remediation applications, with a particular focus on soil amendment and heavy metal pollution control [13]. In recent years (2022–2025), “Cr(VI),” “zero-valent iron,” and “carbonization” have emerged as new hotspots, with research deepening into the targeted remediation of specific pollutants such as hexavalent chromium, the preparation of modified biochar, and the elucidation of its mechanisms of action.

#### 2.3.3. Co-Occurrence Analysis of Countries and Institutions

**Figure 5:**
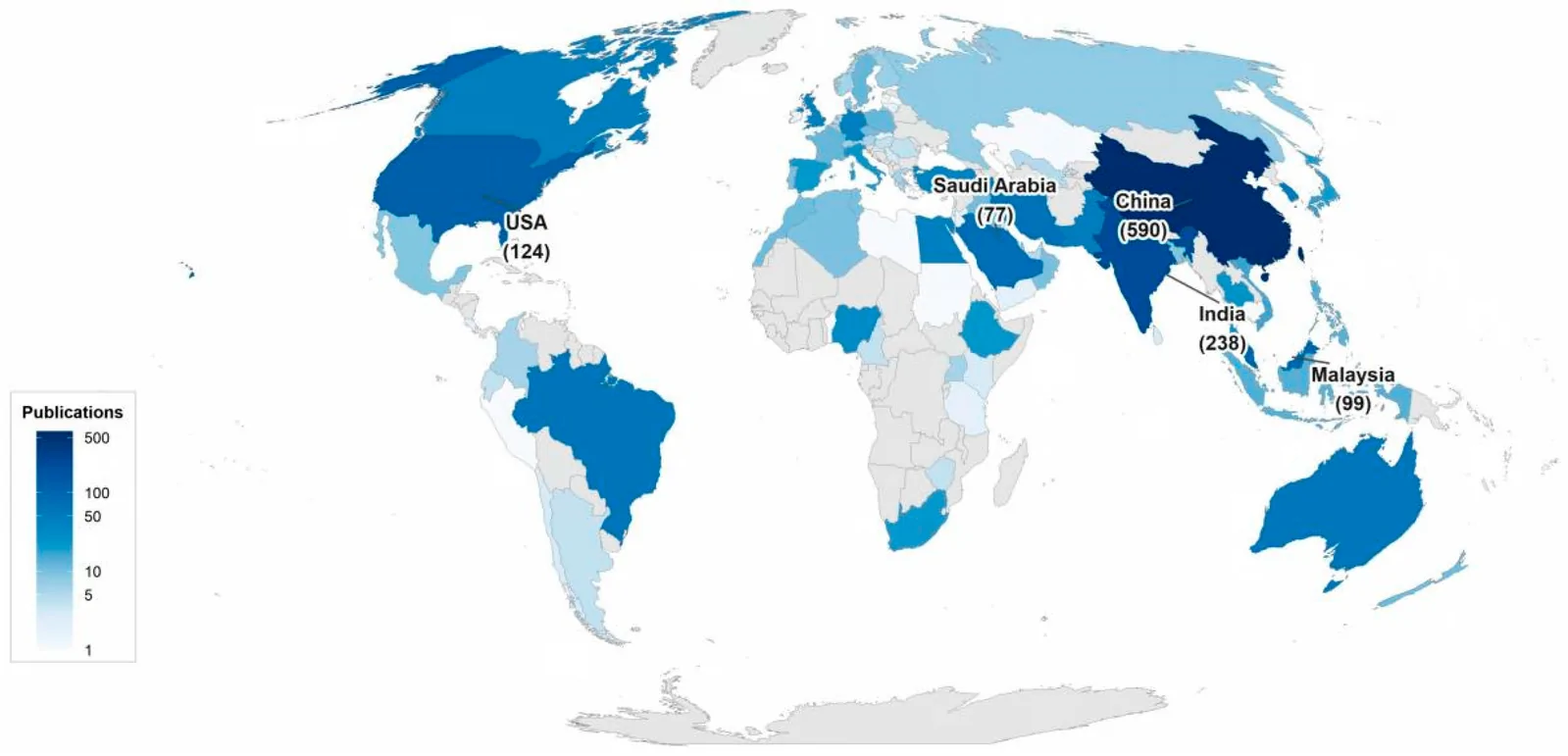
Global geographic distribution of publication counts.

The institutional co-occurrence analysis shows that several stable research teams have formed in this field, with research teams from universities and research institutions such as the Chinese Academy of Sciences, the National Institute of Technology (NIT System), and the Egyptian Knowledge Bank being the most active. The results of global publication counts based on country co-occurrence analysis (Figure 5) indicate that China has the highest number of publications in this field. Because a single publication may be co-authored by institutions from multiple countries, the country-level statistics are reported under full counting of country occurrences, in which each publication is attributed to every country listed in its author affiliations; on this basis, the total number of country occurrences is 2145, and China accounts for 27.5% (590 occurrences), followed by India (11.1%, 238 occurrences), the United States (5.8%, 124 occurrences), and Malaysia (4.6%, 99 occurrences). These results demonstrate that China is at the global forefront in terms of the prevalence of applied research on RSM for optimizing biochar performance.

#### 2.3.4. Synthesis of Bibliometric Findings and Construction of Application Framework

**Figure 6:**
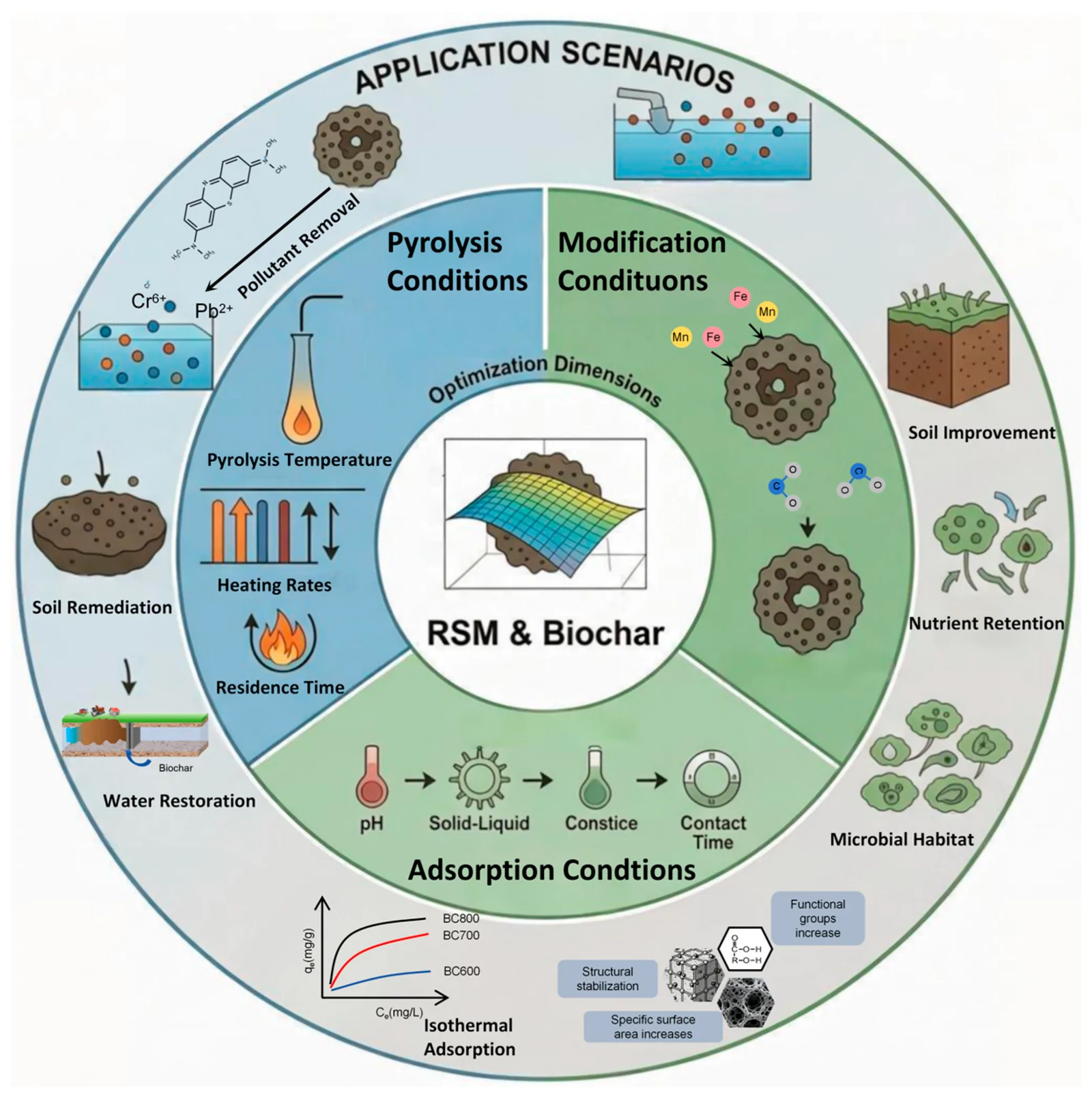
Framework diagram of RSM application in biochar performance optimization.

Synthesizing the above bibliometric findings—including keyword clustering, evolution of burst keywords, and classification of research contents—a comprehensive performance optimization application system centered on biochar was established via RSM. This system covers three core research fields—pollutant adsorption, preparation process regulation, and material modification—and is ultimately applied to water–soil environmental remediation and improvement scenarios. The framework of this application system is presented in Figure 6.

## 3. Basic Principles of RSM and Common Experimental Designs

### 3.1. Basic Principles of RSM

The fundamental concept of RSM is to obtain experimental data on the relationship between factors and response values through a series of well-designed experiments. Multivariate regression analysis is then used to fit a mathematical model that reflects this relationship. Finally, by analyzing and solving this model, the optimal process parameters are determined [14]. The core of this method is to establish a regression equation that accurately describes the relationship between factors and response values; a quadratic polynomial model is typically used, with the general form

(1) $$Y=\beta_{0}+\sum_{i=1}^{k}\beta_{i}X_{i}+\sum_{i=1}^{k}\beta_{ii}X_{i}^{2}+\sum_{i<j}\beta_{ij}X_{i}X_{j}+\varepsilon$$

where Y is the response variable, $X_{i}$ and $X_{j}$ are the independent variables (i.e., influencing factors), $\beta_{0}$ is the constant term, $\beta_{i}$ is the coefficient of the linear term, $\beta_{ii}$ is the coefficient of the quadratic term, $\beta_{ij}$ is the coefficient of the interaction term, k is the number of factors, and ε is the random error.

The coefficients in the regression equation are estimated using the least squares method, followed by significance tests and goodness-of-fit tests to determine whether the model adequately describes the relationship between the factors and the response values. For example, in their study on optimizing the preparation process of water hyacinth straw biochar, Zhou et al. fitted a polynomial regression equation. Analysis of variance (ANOVA) revealed that the model was statistically significant (*p* < 0.0001), with a coefficient of determination R^2^ = 0.9993, indicating that the model provided a good fit to the experimental data [15]. It is important to emphasize that a high R^2^ and a significant model F-test describe the goodness of fit to the observed data and do not, by themselves, demonstrate that the model captures an underlying causal relationship or that it will generalize to new conditions; predictive performance must instead be assessed through independent confirmation experiments, predicted R^2^, cross-validation, or lack-of-fit tests. If the model is statistically significant and provides an adequate fit, it can be used to predict response values under different combinations of factor levels and to determine the optimal combination of factor levels by solving the regression equation. After confirming that the response surface model was significant, Aslam et al. used the model to predict the dye adsorption rates corresponding to various process parameters for iron oxide-modified biochar. They then solved the regression equation to determine the reaction conditions yielding the best performance, and experimental validation showed that the results were generally consistent with the model predictions [16].

Throughout this review, the terms “model fit” and “predictive performance” are used in a strictly distinct sense. Goodness of fit—quantified by the coefficient of determination (R^2^), adjusted R^2^, and the model F-test—reflects how well the model reproduces the observed data used to estimate its coefficients; a high R^2^ alone does not demonstrate predictive accuracy. Predictive performance, by contrast, refers to how well the model generalizes to data not used in fitting, and this should be evaluated using predicted R^2^, cross-validation, lack-of-fit tests, independent confirmation experiments, and reported prediction errors. This distinction is applied consistently when interpreting the studies summarized in Tables.

In biochar performance optimization, the response values are typically performance indicators such as biochar yield, specific surface area, pore volume, adsorption capacity, and cation-exchange capacity, while the influencing factors include pyrolysis temperature, pyrolysis time, heating rate, activator type, activator concentration, activation temperature, activation time, solid-to-liquid ratio, pH, adsorption time, and adsorption temperature [9,17,18]. Through RSM, it is possible to simultaneously examine the effects of multiple factors and their interactions on biochar performance, thereby identifying the optimal process conditions.

### 3.2. Common Experimental Design Methods

There are many experimental design methods within RSM. Among 1468 valid publications, 92.3% of the studies used either a central composite design (CCD) or a Box-–Behnken design (BBD), with CCD accounting for 56.8% and BBD for 35.5%. This is because CCD and BBD are the most classic and easiest-to-implement RSM experimental design methods, and most statistical software (such as Design-Expert and Minitab) provides ready-made templates for them.

#### 3.2.1. Central Composite Design

The central composite design is suitable for optimization experiments with 2–5 factors. It consists of three parts: (1) $2^{k}$ analysis points, located at the vertices of a cube, used to examine the main effects and interaction effects of the factors; (2) 2k axial points, located on the coordinate axes at a distance of α from the center point, used to examine the quadratic effects of the factors; and (3) $n_{c}$ central points, located at the center of the cube, used to estimate experimental error and test the model’s goodness of fit.

The number of experiments in a CCD is $N=2^{k}+2k+n_{c}$, where k is the number of factors, and $n_{c}$ is typically set to 3–5. The selection of α is usually determined based on whether the design is rotational or orthogonal. When $\alpha =(2^{k}{)}^{1/4}$, the design is rotational, meaning that the predicted variance of the response is equal at all points equidistant from the center point, which is highly advantageous for finding optimal values.

In the optimization of biochar production processes, CCD is often used to investigate the effects of factors such as pyrolysis temperature, pyrolysis time, and heating rate on biochar yield and specific surface area. For example, Dzarma et al. used CCD to study the effects of pyrolysis temperature (450–650 °C), pyrolysis residence time (1–3 h), and heating rate (10–20 °C/min) on the biochar yield from baobab shells. They established a quadratic polynomial regression model and, by solving the model, determined the optimal preparation process conditions [19]. Due to its unique advantage of covering extreme variable combinations—the axial points extend beyond the factorial cube while remaining within the planned experimental region—CCD is widely used in biochar optimization studies that require testing under extreme conditions and characterizing the response surface over a broad factor space. It should be noted, however, that this does not imply that a CCD model validates predictions outside the planned experimental region; predictions beyond the axial points remain extrapolations and require independent confirmation.

#### 3.2.2. Box–Behnken Design

The Box–Behnken design is another commonly used response surface experimental design method, suitable for optimization experiments involving 3–5 factors. It is a three-level experimental design in which the design points are located at the midpoints of the cube’s edges and at the center point, excluding the cube’s vertices. The number of experiments in a BBD is $N=2k(k-1)+n_{c}$, where k is the number of factors, and $n_{c}$ is typically set to 3–5.

Compared to CCD, BBD requires fewer experiments. For example, when k=3, a central composite design requires 15–20 experiments, whereas BBD requires only 13–17 experiments. Furthermore, BBD does not require setting high- and low-level combinations of factors, thereby avoiding experiments under extreme conditions, which is particularly advantageous for experiments that are difficult to conduct under such conditions [20].

BBD has also been widely applied in the optimization of biochar production processes. Unlike CCD, which includes axial points that extend beyond the factorial cube, BBD does not require setting high- and low-level combinations of factors at the cube vertices, thereby avoiding experiments under extreme operating conditions. This makes BBD better suited for biochar production scenarios—such as pyrolysis and activation—where conducting multifactor extreme coupled experiments is impractical, making it the mainstream choice for optimizing process parameters with small to medium-sized variable modifications.

#### 3.2.3. Other Experimental Designs

In addition to CCD and BBD, several alternative response surface designs are available and have been applied to a lesser extent in biochar research. The Doehlert (uniform shell) design requires fewer experimental points than CCD for the same number of factors, distributes the points uniformly over the experimental region, and allows the sequential addition of factors or levels to a previously run design, which is advantageous when the factor space is explored incrementally [21]. D-optimal and other optimal (computer-generated) designs select a subset of candidate runs to maximize a chosen information criterion; they are particularly useful when the experimental region is irregular or constrained, when a subset of a larger candidate design must be used, or when categorical factors are present. Sequential screening–optimization strategies combine a first-stage screening design (e.g., a factorial or fractional factorial design) to identify the influential factors with a second-stage response surface design to refine the optimum, thereby reducing the total number of experiments when many candidate factors must first be narrowed down. These designs extend the applicability of RSM beyond the CCD/BBD paradigm that dominates the present corpus (92.3% of the 1468 included publications), and their adoption is recommended when experimental constraints make CCD or BBD inefficient.

Table 3 compares the characteristics and scope of application of CCD and BBD.

## 4. Application of RSM in the Optimization of Biochar Adsorption Performance

Adsorption is the primary application of biochar in environmental remediation, and its adsorption performance is influenced by various factors, such as solution pH and solid-to-liquid ratio. RSM can simultaneously examine the effects of these factors and their interactions on adsorption capacity, thereby identifying optimal adsorption process conditions and improving the adsorption efficiency of biochar (Table 4).

### 4.1. Optimization of Adsorption Performance for Heavy Metal Ions

Heavy metal pollution in water is characterized by high toxicity, high persistence, and high mobility, posing a persistent threat to aquatic ecosystems and human health, and representing a key challenge in the field of environmental remediation. Due to its multifunctional environmental remediation capabilities, biochar is widely used for the adsorption of heavy metal ions. In the RSM model, solution pH is considered to be the primary variable affecting heavy metal adsorption efficiency [34], but its optimal value varies significantly depending on the charge characteristics of the target metal ions.

The adsorption of the cationic heavy metals Cd^2+^ and Pb^2+^ is significantly enhanced under alkaline conditions. Limmun and Bakr et al. confirmed the optimal pH for Cd^2+^ to be 8 using the CCD and BBD models, respectively [23,35], while Ye et al. similarly determined the optimal pH for Pb^2+^ at 8.65 within the CCD framework; an alkaline environment promotes deprotonation of the adsorbent surface, thereby enhancing the electrostatic attraction to positively charged ions [22]. It should be noted, however, that at these alkaline pH values (approximately 8–8.65), metal hydrolysis and the precipitation of sparingly soluble hydroxides may contribute substantially to the apparent removal of Cd^2+^ and Pb^2+^; the enhanced removal should therefore be interpreted as the combined effect of adsorption and precipitation/speciation rather than adsorption alone, and the extent to which the cited studies separated these contributions should be verified before drawing purely adsorptive mechanistic conclusions. The behavior of the anionic Cr(VI) is exactly the opposite: based on the BBD model, Tan found that pH had the greatest influence among the five factors on Cr(VI) removal efficiency, with an optimal pH of only 2; at this pH, protonation of the biochar surface facilitates the electrostatic capture of HCrO_4_^−^ [36]. The response surface optimization results for Hg(II) fall between these two cases; Lim et al. obtained an optimal pH of 3.9 using the BBD model, which is closely related to the fact that Hg(II) exists in cationic form in the pH range but hydrolyzes to uncharged Hg(OH)_2_—a process that results in the loss of electrostatic interactions [24]. Aside from pH, the amount of adsorbent added and the initial concentration of heavy metals constitute another set of key interactive effects: Arabkhani incorporated the initial concentrations of both Co(II) and Pb(II) into a five-factor CCD model, revealing the inhibitory effect of competitive adsorption on removal efficiency in a binary metal system [37]; Limmun’s four-factor CCD model, on the other hand, showed that the interaction between the initial concentration of Cd(II) and pH was far greater than the interaction between concentration and adsorption time [23], suggesting that the influence of metal ion concentration on adsorption efficiency is modulated by the solution’s acid–base environment. There are also significant differences in the combinations of factors included in response surface models across different studies—Hussain selected only three factors (pH, contact time, and dosage) to optimize a multi-metal competitive system [38]; Tan incorporated temperature into a five-factor BBD model and found that Cr(VI) removal efficiency could reach 100% at 45 °C [31]; Ye further included dissolved organic carbon concentration as a third optimization variable and found that low concentrations of humic acid promote Pb^2+^ adsorption, whereas high concentrations inhibit it due to a surface coverage effect [22]; this bidirectional effect is difficult to fully elucidate in traditional single-factor experiments.

### 4.2. Optimization of Adsorption Performance for Organic Pollutants

RSM can be combined with the intrinsic physicochemical characteristics of organic pollutants—such as molecular charge, dissociation properties, and hydrophobicity—to systematically quantify the primary and secondary contributions of multiple environmental factors and adsorbent modification components, as well as the patterns of their interactions and coupling. This provides a quantitative research tool for precisely regulating process parameters and elucidating differentiated adsorption mechanisms in the removal of various types of organic pollutants by biochar-based materials.

The BBD quadratic model for the adsorption of levofloxacin by tea waste biochar incorporated four factors: pH, concentration, dosage, and time. Analysis of variance (ANOVA) revealed that the concentration–time interaction effect and the quadratic terms of each factor significantly influenced the adsorption capacity; the model’s F-value reached 290.59, indicating that the system is highly sensitive to operating conditions [27]. The optimization of phenol adsorption by biochar derived from jujube palm petioles also adopted a multifactorial synergistic approach. Pareto analysis showed that dosage was the dominant factor, with a contribution rate of 34.53%, while pH contributed only 0.25%, indicating that the adsorption of neutral phenol is primarily driven by the solid–liquid ratio and contact time, with electrostatic interactions having a negligible effect [26]. When the adsorbate was expanded from phenolic compounds to drug molecules with different charges, the factor weights shifted significantly: optimization of the BBD for biochar adsorption of atenolol and sulfamethoxazole revealed that pH contributed 24.81% to the removal of sulfamethoxazole, far exceeding its influence on atenolol, consistent with the mechanism whereby the hydrophobic interaction of neutral sulfamethoxazole is enhanced at low pH [39]. Upon introducing ionic strength, the CCD model revealed more complex interaction effects: in the adsorption of four drugs by Toona sinensis leaf-activated biochar, the solid-to-liquid ratio was the most influential linear factor, while the interaction between pH and ionic strength existed only for specific adsorbates, and its promoting or inhibiting effect depended on the direction of electrostatic interactions [28]. Five-factor CCD optimization further increased the complexity of interactions: when sugarcane bagasse biochar–chitosan–zero-valent iron composites adsorbed aspirin and carbamazepine, the inclusion of the temperature factor revealed that the regression coefficients of pH for the removal of these two compounds were −17.12 and 10.09, respectively, with opposite response directions, stemming from the acidic dissociation of aspirin and the neutral charge inertness of carbamazepine [40]. After biochar was combined with layered bi-metallic oxides, the optimal pH conditions underwent a fundamental shift: the optimal pH for phenol removal by magnetic biochar/Ca-Al-FeLDO composites was 2.0 rather than near-neutral; the positive charge sites introduced by LDO made complexation and ion exchange the dominant mechanisms under acidic conditions, demonstrating the role of modification in reshaping the scope for optimizing operating conditions [41].

### 4.3. Optimization of Adsorption Performance for Non-Metallic Inorganic Compounds

Anionic non-metallic inorganic pollutants possess unique characteristics, such as charge and a speciation that varies dramatically with pH, and adsorption that is highly dependent on the protonation structure at the material interface [42]. The principle of quantifying and regulating nonlinear relationships through RSM demonstrates unique analytical capabilities in the adsorption of non-metallic inorganic anions.

Mazarji et al. used CCD to jointly model the GMBC dosing ratio and solution pH; ANOVA revealed that both the pH squared term and the dosing ratio × pH interaction term were significant, indicating that at low pH, the marginal effect of the dosing amount on nitrate removal increases as pH decreases [31]. Tan et al.’s CCD optimization, however, revealed a different form of interaction structure: the “HCl concentration × heating time” interaction term was significant, while the “HCl concentration × heating temperature” interaction term was not. This suggests that protonation and thermal activation effects act independently rather than interdependently, and that the significance of interaction terms directly determines the degree of distortion in the response surface and the optimization search path [43]. Zhou et al.’s BBD contribution analysis revealed a deeper regulatory logic behind RSM: The effects of biochar dosage (46.43%) and calcination temperature (37.55%) on phosphate adsorption far exceeded those of the Mg/Al molar ratio (16.02%), and the single-factor correlations between these three preparation parameters and adsorption capacity were extremely weak, whereas the interlayer spacing showed a significant positive correlation with adsorption capacity (R = 0.83) [44]. This implies that RSM does not regulate the direct mapping of variables to the response but, rather, the indirect control of performance by structural mediating parameters induced by combinations of variables. The negative coefficients for pyrolysis temperature and Fe concentration in Kang et al.’s regression model indicate that the RSM quadratic term captures the non-monotonic relationship of decreasing response beyond a threshold; the finding that the amorphous Fe phase at 300 °C outperforms the high-temperature crystalline phase precisely corresponds to the optimization boundary indicated by the inflection point on the response surface [30]. Li et al. combined machine learning with RSM; the response surface topologies for the three types of biomass were strikingly different—all three factors were significant for rice straw, whereas La^3+^ concentration was the sole dominant factor in walnut shells and bamboo, indicating that the same optimization framework converges to completely different variable weight configurations across different feedstock systems [45].

## 5. Application of RSM in the Optimization of Biochar Production Processes

The biochar preparation process is a key factor determining its performance, with pyrolysis temperature, pyrolysis time, and heating rate being the three most influential parameters. Pyrolysis temperature affects the pore structure and surface chemical properties of biochar; as the pyrolysis temperature increases, the specific surface area and pore volume of biochar gradually increase, but the yield gradually decreases [46]. Pyrolysis time affects the degree of carbonization of biochar; if the pyrolysis time is too short, the biomass will not be fully carbonized, while if the pyrolysis time is too long, the pore structure of the biochar will collapse [47]. The heating rate affects the pore distribution of biochar; an excessively fast heating rate leads to rapid pyrolysis of the biomass, producing a large amount of volatile matter and thereby forming more micropores, while an excessively slow heating rate results in a more developed pore structure but with larger pore sizes [48].

RSM can simultaneously examine the effects of these three factors and their interactions on biochar performance, thereby identifying the optimal preparation process conditions. Depending on the type of feedstock, the application of RSM in optimizing biochar preparation processes can be categorized into plant-based biochar, animal-based biochar, and sludge-based biochar [49]. The application results are shown in Table 5.

### 5.1. Optimization of the Plant-Based Biochar Production Process

Plants are the primary source of raw materials for biochar, including agricultural waste such as crop straw, rice husks, corn cobs, and peanut shells, as well as forestry waste such as wood chips, bark, branches, and leaves [59,60,61]. These raw materials are widely available, produced in large quantities, and inexpensive, and they are rich in cellulose, hemicellulose, and lignin, making them suitable for the production of high-performance biochar.

BBD is suitable for investigating the fundamental law of biochar yield. By constructing a quadratic prediction model for the corn cob pyrolysis system, it was determined that the effects of pyrolysis temperature, holding time, and heating rate on yield decrease progressively, with temperature being the core controlling factor. Based on this model, differences in raw material responses can also be distinguished: corn leaves with high ash and lignin contents are more sensitive to holding time, and under self-purge pyrolysis conditions, the optimal yield can reach 37.91% [50]. Compared to the single-criterion optimization of BBD, CCD achieves higher-precision multi-objective optimization, expanding the evaluation scope from yield to fuel quality indicators such as calorific value and energy density. This RSM model was used to optimize the pyrolysis process. Yadav et al. produced rice husk biochar of near-lignite quality under conditions of 432 °C, 40 min, and 4 °C/min, and revealed trade-offs among pyrolysis parameters: temperature improves fuel quality but reduces yield, while holding time and heating rate synergistically influence carbon and energy retention by regulating the escape of volatiles [51]. Furthermore, a combined study of RSM fitting and pyrolysis kinetics enabled the precise quantification of the interaction between temperature and heating rate in a peanut shell pyrolysis system. This confirmed that high temperatures and high heating rates intensify the thermal cracking of cellulose and reduce charcoal yield; this coupling mechanism can be accurately characterized using a quadratic RSM model, providing a quantitative basis for the precise multi-objective control of biochar pyrolysis [62].

Compared to agricultural waste, which is characterized by high compositional heterogeneity and significant fluctuations in ash content, forestry woody feedstocks feature a uniform fiber structure and low ash content; consequently, their pyrolysis parameter response mechanisms and optimal control ranges exhibit distinct patterns. Using pruned apple tree branches from forestry operations as feedstock, Yang et al. expanded the optimization objectives to the dual dimensions of yield and fixed carbon, revealing the significant effects of the temperature–retention time interaction and clarifying the parameter trade-off range for low-ash lignocellulosic feedstocks [63]. Paudel et al. obtained the globally optimal pyrolysis control range for oak sawdust biochar through multi-objective optimization, achieving a carbonization rate of 95.26% and an absolute yield of 37.31%, demonstrating a wider stable process window compared to high-ash agricultural straw [64].

### 5.2. Optimization of the Production Process for Animal-Based Biochar

Animal-based feedstocks consist primarily of livestock and poultry manure, which exhibits the characteristics of biomass that is both high in nitrogen and rich in mineral ash; consequently, the logic for controlling the pyrolysis process differs from that for lignocellulosic feedstocks. The large amount of inorganic ash in manure catalyzes the decomposition of organic matter and clogs newly formed pores, while nitrogen components simultaneously participate in the cross-linking reaction to form biochar. Parameter control must therefore balance the catalytic effect of ash with the retention of nitrogen-containing functional groups, whereas for lignocellulosic feedstocks, the focus is solely on the balance between carbon skeleton pyrolysis and pore formation. CCD can precisely quantify the coupled effects of multiple pyrolysis parameters and is the mainstream optimization method for the targeted production of manure-based biochar.

Based on CCD, and using biochar specific surface area as the response variable, Yıldız et al. conducted a multifactorial coupled optimization of temperature, heating rate, and reaction time for the fluidized-bed pyrolysis of chicken manure. The optimized operating conditions were determined to be 400 °C, 5 °C/min, and 110 min, with a model R^2^ = 0.8717, indicating a good fit to the experimental data; however, this goodness of fit does not by itself establish predictive accuracy, which would require independent confirmation [54]. To elucidate the impact of equipment structure on RSM optimization results, previous studies extended the optimization approach to fixed-bed systems and found a significant shift in the optimal process parameters. The optimal conditions were 440 °C, 7 °C/min, and 115 min, with the specific surface area of biochar increasing to 4.61 m^2^/g. Analysis of RSM parameter weights reveals that pyrolysis temperature consistently remains the dominant factor in controlling pore structure, while the relative contributions of heating rate and residence time vary with changes in the reactor’s mass transfer characteristics, indicating that reactor configuration directly alters the optimal RSM parameter range [53]. A comparison of the RSM optimization results shows that the fluidized bed exhibits more significant advantages in heat and mass transfer, enabling equivalent charcoal production at lower temperatures; therefore, laboratory RSM optimization parameters cannot be directly scaled up for engineering applications, and parameter deviations must be corrected based on the reactor type. To address the issues of high ash content in single-source manure and limited pore development, the study extended the application of RSM to a co-pyrolysis system of cassava peel and sheep manure, with charcoal yield as the response variable for parameter optimization. Experiments showed that temperature exerts a far stronger regulatory effect on the co-pyrolysis process than residence time; a 1:1 mixture of raw materials achieved a charcoal yield of 93.2% under conditions of 237.87 °C and 37.5 min. The optimal parameter matching achieved through RSM effectively harnessed the synergistic effects of the raw materials, thereby addressing the shortcomings of pyrolysis using manure alone [55].

### 5.3. Optimization of the Sludge-Based Biochar Production Process

Sludge feedstocks vary widely in origin, with significant differences in organic matter and ash content. Due to variations in feedstock matrix, evaluation criteria, and coupled processes, the optimized pyrolysis systems for sludge-based biochar from different sources exhibit distinctly different optimal pyrolysis ranges and patterns of variable interactions. There are generally trade-offs and constraints among the multidimensional responses of performance, yield, and environmental risks.

When preparing uranium-adsorbing biochar from slaughterhouse sludge and rice husk fermentation products, a BBD combined with a BP neural network—using the U(VI) removal rate as the response variable—determined that 382 °C was the optimal pyrolysis temperature, and that the interaction effect between temperature and holding time was the most significant [57]. Although the optimization of palm oil mill sludge biochar for SO_2_ removal also confirmed that temperature is the dominant variable, the optimal temperature was only 405 °C [65]. This discrepancy reflects how the organic composition of different sludges profoundly shapes the temperature response window. Studies on the promotion of anaerobic methane production using biocarbon derived from digested sludge shifted the response variable to functional application performance; using methane yield as the response via CCD, the optimal temperature rose to 516 °C [56]. This relatively high temperature threshold is closely related to the need to retain abundant surface oxygen-containing functional groups after deep carbonization of digested sludge. When the optimization objectives were expanded to include both yield and CO_2_ adsorption capacity, the response surface of municipal sludge pyrolysis revealed a lack of synchrony between the two—yield decreased with increasing temperature, while adsorption capacity first increased and then decreased [58]. This trade-off stems from the diluting effect of the sludge’s high ash content on the adsorption activity of the carbonaceous material. The design of co-pyrolysis of sludge and wood waste further expanded the optimization space to the feedstock ratio domain, introducing sludge proportion and type as categorical variables. Partial least squares (PLS) regression analysis of multiple responses—including ash content, specific surface area, and polycyclic aromatic hydrocarbon (PAH) release [66]—revealed a contradictory effect: while an increased sludge proportion raises ash content, it reduces PAH release, highlighting the intrinsic balance between environmental safety and product performance in the optimization of sludge-based biochar.

## 6. Application of RSM in the Optimization of Biochar Modification Processes

Unmodified raw biochar typically suffers from drawbacks such as low specific surface area, underdeveloped pore structure, and a limited number of surface functional groups, which limit its application in fields such as environmental pollution remediation. To improve the performance of biochar, it is usually necessary to subject it to modification treatments. Common biochar modification methods include chemical modification, physical modification, and microbial loading. RSM has been widely applied in the optimization of biochar modification processes, effectively optimizing modification parameters and enhancing modification outcomes (Table 6).

### 6.1. Optimization of the Chemical Modification Process

Chemical modification involves mixing biochar with a chemical activator and subjecting the mixture to activation treatment at high temperatures, which can significantly increase the specific surface area and pore volume of the biochar. Chemical activation is characterized by multiple parameter dimensions and strong variable coupling; the regulation of pore structure and surface chemical properties depends on the synergistic effects of multiple process conditions. Common chemical activation methods include acid–base modification and metal modification.

In alkali-modified systems, the interactive effect between KOH concentration and pyrolysis temperature is particularly pronounced: optimization of KOH activation of coconut shell waste biochar revealed that carbonization temperature is the primary controlling factor for iodine value and methylene blue adsorption, while KOH concentration enhances surface reactivity and pore volume through chemical leaching [68]; KOH activation of pulp sludge biochar further indicates that the combined contribution of the activator ratio and temperature to the specific surface area exceeds 60%, with both factors synergistically driving the differentiated development of microporous and mesoporous structures [67]. For NaOH-modified jute fiber biochar, temperature is also the dominant factor; however, the positive effect of NaOH impregnation on specific surface area is independent of temperature, indicating that the mechanism by which the alkaline modifier opens pore channels through saponification is relatively independent [69].

The acid-modified system exhibits distinctly different factor weights. H_3_PO_4_: When activating birch polypore residue biochar, the mass ratio of phosphoric acid to feedstock becomes the most significant linear factor; the cross-linking protective effect of phosphoric acid reduces the optimal pyrolysis temperature from 547 °C for ZnCl_2_-activated biochar to 374 °C [70]. H_2_SO_4_: Optimization of sulfonated sugarcane bagasse biochar revealed that the acid-to-char ratio is the factor with the strongest positive effect on sulfonic acid density; the interaction term between temperature and acid ratio was significant, while the direct effect of reaction time was weak and only became apparent when coupled with other factors [78].

Response surface characteristics in metal-modified systems are more complex: in aluminum-impregnated food waste biochar, the interaction term between pyrolysis temperature and aluminum concentration reached a significant level; aluminum impregnation confers specific adsorption capacity for phosphates on the biochar through a ligand-exchange mechanism [71]. In the case of iron–zinc co-modified coconut shell biochar, it was found that in single-metal systems, metal loading was the primary driver of BET surface area, whereas in bi-metallic systems, heat treatment duration emerged as the most significant variable; the synergistic effect of Fe^3+^ and Zn^2+^ enabled the optimal surface area to exceed 2100 m^2^/g [79]. The redistribution of factor weights in the bi-metallic system implies that the chemical interactions between the modifiers themselves have become a non-negligible latent variable in the response surface.

### 6.2. Optimization of the Physical Modification Process

Physical modification is a method that involves reacting biochar with activators such as CO_2_ and water vapor at high temperatures to etch the carbon framework and form a pore structure. Compared with chemical activation, physical activation offers advantages such as a simple process, low cost, and no chemical pollution; however, it requires a longer activation time, and the resulting biochar has a relatively low specific surface area. The physical activation process of biochar is regulated by the coupled effects of multiple parameters, including temperature, residence time, and activator flow rate. There is a strong nonlinear trade-off between pore evolution and product yield, making RSM widely applicable in this context.

For CO_2_ physical activation systems, BBD is widely adopted because it does not require the setting of extreme operating conditions. Rashidi et al. used palm kernel shell biochar as a precursor and established a dual-response model for yield and CO_2_ adsorption capacity via BBD. They identified activation temperature as the most significant controlling factor; its negative effect on yield and positive effect on adsorption capacity constitute the core trade-off of the process. Ultimately, a dual-objective balance was achieved under conditions of 950 °C, 60 min, and 150 mL/min [73]. Shah et al. applied BBD to the CO_2_ activation of biochar derived from waste disposable diapers. The quadratic model that they constructed achieved a coefficient of determination of 0.9972, accurately capturing the negative interaction between temperature and flow rate—high temperatures at high flow rates tend to cause overactivation, leading to pore collapse—and ultimately yielding a specific surface area of 1235.32 m^2^/g [74].

In steam activation systems, CCD is more suitable for fitting nonlinear responses across a wide range of parameters. Jha et al. used flaxseed meal biochar as a test material and, through CCD-RSM, confirmed that temperature makes the dominant contribution to pore development (F-value 7576.41), while the main effect of activation time was not significant; its influence was only reflected through interaction terms in the high-temperature range, thereby correcting the misconception that “extending activation time inevitably increases specific surface area” [75]. Lopes et al. used a rotatable CCD to optimize the entire steam activation modification process. They selected three core process variables—activation temperature, activation duration, and steam flow rate—and constructed a second-order polynomial model using BET specific surface area and carbon product yield as dual response indicators. After conducting an ANOVA significance test, they determined the optimal modification process [80].

### 6.3. Optimization of the Microbial Loading Process

RSM overcomes the limitation of single-factor experiments, which can only capture the effects of independent variables, and accurately analyzes the nonlinear interactions among multiple factors within the microbial–biochar composite system. It provides a quantitative basis for optimizing the entire process of carrier preparation and microbial immobilization. The second-order polynomial model constructed by RSM quantifies the interactive coupling effects of multiple variables, such as pyrolysis temperature, bacterial suspension dosage, and system temperature.

A BBD study, which incorporated pyrolysis temperature, bacterial suspension dosage, and loading time into the model and used pollutant removal rate as the response variable, revealed a key paradox: although maize straw biochar produced at 500 °C exhibited lower adsorption capacity for imidazole nicotinic acid than the 700 °C sample, its more suitable pore structure and hydrophilic surface made it the optimal carrier for microbial colonization, indicating that high adsorption performance does not necessarily translate into high loading efficiency [76]. Similarly, a study using BBD to examine the effects of experimental temperature, biochar dosage, and pyrolysis temperature on phenol removal efficiency further confirmed that pyrolysis temperature is the dominant factor; wheat straw biochar derived from demineralized straw at 800 °C achieved optimal synergy between microbial attachment and degradation due to its high specific surface area and degree of graphitization. However, both studies used pollutant removal efficiency as the response variable, effectively treating microbial loading as an intermediate step in the degradation system rather than an independent optimization target [81].

In contrast, a study using CCD with Bacillus colony count (CFU/mL) as the response variable directly anchored the optimization objective to microbial loading density itself. By incorporating four operational factors—temperature, rotation speed, pH, and starch addition—the study found that the introduction of a co-carbon source (sago starch) significantly enhanced microbial attachment and survival on oil palm shell biochar by promoting the production of extracellular polymers and biosurfactants [77]. This marks a paradigm shift in response surface optimization from the indirect logic of “load serving degradation” to the direct logic of “the load itself as the objective.”

## 7. Current Issues and Future Prospects

This section synthesizes the main limitations of current RSM-based biochar optimization studies and the corresponding future prospects. The limitations are organized around four themes: (i) the fitting capability of quadratic polynomial models and the associated risk of nonlinear response misspecification; (ii) the gap between goodness of fit and predictive performance; (iii) the scarcity of multi-response collaborative optimization and the difficulty of balancing competing objectives; and (iv) insufficient model validation and extrapolation risks. For each limitation, the corresponding future direction is outlined, followed by a proposal for an integrated framework that couples RSM experimental design with machine learning.

### 7.1. Existing Issues

#### 7.1.1. Limitations of Quadratic Polynomial Model Fitting and Nonlinear Response Distortion

A core assumption of RSM is that the relationship between factors and response values can be approximated by a quadratic polynomial; however, many response behaviors during biochar preparation and modification exhibit significant higher-order nonlinear characteristics, resulting in fundamental limitations in the fitting capability of quadratic models.

In research on soybean straw biochar, the adjusted R^2^ of the pH model was only 0.9219, and the *p*-values for the interaction terms AB and BC, as well as the linear term B, were all equal to 1. This suggests that the quadratic structure may not fully accommodate the actual variation pattern of pH [82]. Forcing the inclusion of numerous statistically insignificant terms can lead to structural mismatch, because a non-quadratic response is being fitted to a parabolic template. Yusuff et al.’s study on Cr(VI) adsorption provides another example: the adjusted R^2^ of RSM was only 0.7214, compared with 0.8908 for an adaptive neuro-fuzzy inference system (ANFIS)—a difference of 0.1694. This indicates that a substantial share of the response variability was not captured by the parabolic assumption; the quadratic model could only approximate the function’s behavior near the optimal point and did not faithfully reproduce the true trend of the response surface when far from the optimal region [83]. Although ul-Ain et al. obtained a higher adjusted R^2^, the initial concentration term and several interaction terms in their quadratic model were not significant, whereas the ANN achieved higher accuracy with fewer constraints (adjusted R^2^ reached 0.998). This indicates that even with an acceptable overall fit, the quadratic model may not fully capture local nonlinear features and instead absorbs them into redundant terms [84].

It is worth noting that the degree of model limitation is not uniformly distributed: the R^2^ reached 0.966 for one response, whereas the pH model achieved only 0.9219 [82], and the HBC model of ul-Ain et al. outperformed the H model [84]. This suggests that a quadratic fit is more reliable when the response surface is close to a symmetric parabolic shape; however, when the true curvature exhibits asymmetry, multiple extrema, or gradual inflection points, a quadratic polynomial may force the projection of nonlinear features into quadratic space, at the cost of non-significant terms and a decline in R^2^. These findings indicate that apparent goodness of fit and the adequacy of the quadratic approximation should be evaluated separately, and that a lower R^2^ or non-significant terms are best interpreted as indicators of possible model misspecification rather than as definitive evidence of a specific nonlinear distortion.

#### 7.1.2. Monotonicity of Experimental Design Methods and Design Space Constraints

In current research on biochar performance optimization, RSM-based experimental designs exhibit a strong tendency toward uniformity. The vast majority of studies use only CCD or BBD as the sole tool for constructing experimental designs, with few integrating screening strategies such as Taguchi designs into the RSM system. CCD and BBD provide a second-order fitting framework with a relatively small number of experiments, at the cost of rigid constraints on the design space—CCD axial points often fall outside the operational range, while BBD is limited to three levels and lacks extreme points, resulting in significantly degraded prediction accuracy at the endpoints. The set of experimental data points generated by RSM is fixed, and subsequent modeling can only be performed by fitting these predetermined points; the “optimal solution” sought is essentially constrained by the artificial boundaries defined in the initial design rather than representing a global optimum [11].

Xie et al. constructed three independent CCD models for the specific surface area, conductivity, and pH of soybean straw charcoal, respectively [82], revealing a structural dilemma in RSM-based multi-response collaborative optimization: when the optimal conditions for different responses point to different combinations of process parameters, a single model cannot accommodate such conflicts. Vikraman’s research demonstrated the fragility of preset design spaces—heating rates of 5–15 °C/min had no significant effect on fixed carbon, pH, or electrical conductivity [85], as the selected factor levels were too narrow to cover the actual range of effects. Roman’s multipath comparison further indicated that the RSM quadratic model did not achieve statistical significance under air gasification conditions [7], and that the simple quadratic surface assumption was insufficient to capture the higher-order interaction effects between biomass and temperature in an oxygen-containing atmosphere. While RSM aims to improve experimental efficiency, it does so at the expense of design space flexibility and the model’s ability to characterize complex response behavior; this fundamental contradiction has not yet been fully examined in existing research.

#### 7.1.3. Simplification of Multi-Objective Optimization and Lack of Trade-Off Mechanisms

Existing RSM-based studies on biochar optimization often simplify complex multi-objective systems into single-response optimization. Tibebu et al. used biochar yield as the sole optimization criterion, determining optimal pyrolysis conditions by maximizing yield through an objective function [9]. However, yield is significantly traded off with functional indicators such as fixed carbon content and specific surface area: low-temperature, short-duration conditions favor higher yields but inhibit the formation of aromatic structures and pore development in biochar. Although the feasibility of single-yield optimization can be verified based on specific application scenarios, the lack of coordinated regulation among mutually conflicting performance objectives reduces the multidimensional process optimization problem to a unidimensional optimization search.

To overcome the limitations of single-objective optimization, Rabbi developed a multi-objective optimization framework combining multi-output random forests with the differential evolution algorithm. By simultaneously optimizing biochar specific surface area, CO_2_ adsorption, specific capacitance, and carbon stability via the Pareto front, the study identified a trade-off between carbon stability and specific capacitance: samples with a stability index exceeding 0.6 exhibited only moderate specific capacitance, making it difficult to achieve simultaneous optimization of both metrics [86]. This indicates that multi-objective trade-offs cannot be resolved through simple compromises in process parameters; rather, a quantification mechanism must be established to clarify the extent of trade-offs between metrics. At the same time, the prediction accuracy for carbon stability is only R^2^ = 0.497, with significant prediction errors for core metrics, which undermines the reliability of the Pareto front in the stability dimension.

Consequently, single-objective optimization obscures the trade-offs between metrics, making it difficult to balance overall performance; while multi-objective optimization can explicitly characterize these trade-offs, the theoretical and data support for quantifying multi-objective trade-offs remains insufficient due to the predictive accuracy limitations of surrogate models.

#### 7.1.4. Insufficient Model Validation and Extrapolation Risks

The evaluation of RSM model fit relies solely on metrics within the design space and lacks reliability tests for out-of-domain extrapolation, making it prone to the problem of precise in-domain fitting but failed out-of-domain predictions.

Response surface models exhibit extremely high interpolation accuracy within the experimental domain, with prediction errors below 1%; however, under the same operating conditions, machine learning models exhibit significant and systematic prediction biases, with errors concentrated in the range of moderate operating conditions. This indicates that the high fit of the response surface quadratic polynomial is merely a local mathematical fit based on limited experimental data and does not align with the physical mechanisms of biochar production; its response surface deviates significantly from actual operating conditions outside the experimental parameter range [87].

Research on multifactor models further corroborates this shortcoming: while the model can keep errors within 5% within the experimental range, errors surge to 18.52% when extrapolating across literature data. Some key process parameters show no statistical significance within the narrow experimental range; the model’s response estimates for these parameters lack data support, and extrapolations beyond the domain are based on unverified assumptions, resulting in insufficient reliability [88].

Under conditions of strong nonlinearity, the limitations of response surface models become even more apparent. Their underlying linear assumptions cannot accommodate complex variable interactions; even if they pass variance significance tests, their out-of-domain generalization capability is extremely poor due to the narrow study range of experimental parameters, formulations, and loads, and their fitting accuracy is far lower than that of machine learning models [89,90].

Therefore, the high goodness-of-fit and low residuals of response surface models only demonstrate their in-domain interpolation capabilities; they lack reliable predictive performance across different conditions, scales, and raw materials. The current general paradigm of relying solely on data within the experimental domain to validate model credibility avoids a systematic assessment of extrapolation risks and, thus, contains validation flaws.

### 7.2. Future Prospects

#### 7.2.1. Establishing an Integrated Framework Combining RSM Experimental Design and Machine Learning

Currently, the combined application of RSM and ML largely remains at the level of “parallel comparison”—using RSM to design experiments, using machine learning to predict responses, and then comparing accuracy [91,92,93]. While this confirms the advantages of ML in nonlinear fitting, it fails to unlock the synergistic potential of the two approaches. RSM excels at revealing factor interactions and response surface shapes, while ML excels at mining key driving factors from high-dimensional data; the two complement each other precisely at different levels of information extraction. Integrated systems should break away from the fixed sequence of “RSM first, ML second” and adopt a sequential strategy of “ML for initial screening, RSM for subsequent refinement”: first, use ML to identify dominant variables and parameter ranges from big data; then, use RSM to perform fine-tuned optimization within the narrowed factor space, thereby significantly reducing the number of experiments. For example, Han et al. integrated random-forest machine learning with RSM to drastically reduce the need for numerous single-factor and multi-gradient trial-and-error experiments. Based on a dataset of 762 biochar samples, they constructed a high-precision prediction model with a test R^2^ of 0.98, and they were able to determine the optimal preparation conditions after validation with only 17 sets of magnesium-modified biochar BBD experiments [94]. Furthermore, a one-size-fits-all approach to model selection is inappropriate, as different algorithms exhibit significant differences in their adaptability to data features [91]; therefore, dynamic model selection criteria tailored to biochar systems must be established. Only by deeply coupling the experimental design logic of RSM with the data-driven capabilities of ML can a new optimization paradigm be established that combines interpretability with high accuracy.

#### 7.2.2. Establishing a Multi-Objective, Multi-Constraint Collaborative Optimization Framework

The coupling of RSM and ML enables the simultaneous optimization of multiple responses—such as specific surface area, electrical conductivity, and pH—within a single framework [82]. Furthermore, the introduction of multi-objective genetic algorithms (MOGAs) and multi-criteria decision-making (MCDM) further addresses the decision-screening problem involving conflicting objectives on the Pareto front [95]. This indicates that multi-objective optimization is no longer limited to algorithmic solution-finding but is evolving toward an integrated “optimization-decision” approach. The engineering application of biochar is inherently subject to multiple constraints—the trade-off between yield and adsorption capacity must be resolved within energy consumption budgets, pollutant leaching limits constitute hard environmental constraints [96], and the cost boundaries of large-scale production determine the feasibility of the optimized solution. In addition to setting constraints around the range of preparation parameter values, the model should further incorporate practical constraints such as energy consumption costs, environmental safety thresholds, and economic feasibility. Therefore, it is necessary to establish an optimization framework that integrates multiple performance objectives and practical constraints: using intelligent algorithms to drive Pareto solutions and MCDM to facilitate preference-based decision-making, while incorporating energy consumption, environmental compliance, and economic indicators into the model as constraint boundaries. This ensures that optimization results not only meet performance maximization requirements but also possess engineering feasibility and environmental compliance, thereby truly bridging the gap between laboratory optimization and engineering applications.

#### 7.2.3. Establishing a Full-Chain Optimization Paradigm and a Sustainable Optimization System

Current RSM optimization focuses on individual stages; in the future, a full-chain integrated optimization paradigm spanning “raw material selection, preparation processes, modification treatments, application performance, and environmental effects” should be established. With end-use performance as the ultimate response variable, upstream parameters should be transmitted to the end response via a cascaded model. Drawing on a three-level progressive optimization strategy, the full chain can be decomposed into multi-level sub-problems, and global optimality can be achieved through inter-level feedback iteration [97]. Full-chain optimization should also incorporate the time dimension—the aging effects of biochar during long-term application will cause the initial performance metrics to gradually deviate from the actual results, necessitating the establishment of a time-dependent RSM model that includes aging kinetics [98]. Furthermore, Life Cycle Assessment (LCA) should be integrated into the RSM framework to construct a “technical performance–environmental impact–economic cost” three-dimensional optimization objective system, using LCA indicators such as GWP, AP, and EP as response variables or constraints. Through multi-objective optimization, the Pareto front between technical performance and environmental impact should be identified, and environmental safety attributes should be set as hard-constrained response variables with regulatory thresholds [99], thereby driving the transformation of biochar research from “materials science focused on pursuing extreme performance” to “systems science balancing technical, environmental, and economic objectives.”

## 8. Conclusions

By combining bibliometric analysis with a systematic review, this paper comprehensively traces the development history, research hotspots, and application frameworks of response surface methodology (RSM) in the field of biochar performance optimization. It clarifies the applicability boundaries of different experimental design methods, reveals the patterns of parameter control across three major scenarios—preparation, modification, and adsorption—and identifies the core limitations and future directions in this field.

Research indicates that the application of RSM in the biochar field has undergone three stages: inception, rapid growth, and stable growth. Currently, three core research clusters have emerged: optimization of adsorption performance, control of preparation processes, and upgrading of modification technologies. In terms of practical value, RSM not only significantly reduces the number of experiments and improves the efficiency of parameter optimization but also enables the quantitative analysis of nonlinear coupling effects among multiple factors through significance analysis of interaction and quadratic terms, thereby addressing the methodological shortcoming of traditional single-factor experiments, which cannot identify synergistic effects.

This paper further distills three conclusions: First, the selection of RSM experimental designs follows clear rules for scenario adaptation—Box–Behnken designs, which do not require the setting of extreme operating conditions, are better suited for preparation and modification scenarios with limited operational boundaries, such as pyrolysis and chemical activation; central composite designs, whose axial points extend beyond the factorial cube while remaining within the planned experimental region, are more suitable for systems requiring the characterization of a wide parameter space, such as adsorption process optimization. These two approaches are not interchangeable universal solutions. Second, RSM can generate hypotheses about factor interactions rather than directly identifying mechanisms: the curvature of the response surface and the significance of interaction terms can indicate which factor interactions most strongly shape the response and can guide the design of complementary experiments, but they do not by themselves establish a dominant adsorption mechanism. For example, a significant pH–initial-concentration interaction may prompt further investigation of speciation, surface charge, and precipitation effects, which must be confirmed using independent characterization and modeling rather than inferred from the response surface alone. Third, the field currently faces a common challenge of good fit within the fitted domain but uncertain generalization outside it. While quadratic polynomial models can achieve prediction errors below 5% within the experimental range, errors can exceed 18% when extrapolating across different feedstocks or scales; simply improving the goodness of fit cannot fundamentally resolve the issue of insufficient generalization.

Overall, RSM remains a core tool for optimizing biochar performance; however, its future development must transcend the limitations of single quadratic models and transition toward a hybrid paradigm of “machine learning for initial screening combined with response surface modeling for fine-tuning.” Concurrently, a multi-objective, end-to-end optimization framework must be established to advance biochar R&D from laboratory-scale performance optimization toward engineering-level system optimization.

## Figures and Tables

**Table 1:** Detailed clustering information for keywords.

Cluster ID	Cluster Label	Count	Cluster Score	Primary Keywords	Main Research Areas
0	Adsorption	92	0.48	Adsorption; kinetics; methylene blue; artificial neural network	Study on adsorption materials and adsorption kinetics
1	Slow pyrolysis	71	0.412	Slow pyrolysis; Box–Behnken design; pyrolysis; biomass; co-pyrolysis	Research on biomass pyrolysis technology
2	Biodiesel	64	0.533	Biodiesel; transesterification; anaerobic digestion; magnetic catalyst	research on biodiesel production and catalytic technologies
3	Response surface methodology	53	0.425	Response surface methodology; plant growth; root morphological traits; sustainable agriculture; soil management	A study on the application of response surface methodology in agriculture and soil management
4	Microbial community	53	0.44	Microbial community; drought tolerance; soil amendments; bacterial community	Research on soil microbial communities and soil amendment technologies
5	Black carbon	48	0.739	Black carbon; charcoal; carbon sequestration	a study on the carbon sequestration effects of black carbon and biochar
6	Water treatment	42	0.556	Water treatment; phosphate removal; phosphorus; wastewater treatment	Research on water treatment and phosphorus removal technologies for wastewater
7	Response surface method	32	0.731	Response surface method; hydrothermal carbonization; persulfate activation; biochar material	A study on the application of the response surface method in biochar materials and environmental catalysis
8	Copper removal	26	0.722	Copper removal; lead; antioxidants; carbon paste electrode; physiology	Research on heavy metal ion removal and electrochemical technologies
9	Dissolved organic carbon	21	0.895	Dissolved organic carbon; retention; kinetic model; pollutants; photocatalysis	Study on the photocatalytic degradation of dissolved organic carbon and pollutant control
10	Hard-setting	18	0.896	Hard-setting; saline soil improvement; carbon mineralization; soil microbial biomass	Research on saline soil improvement and soil carbon cycling processes

**Table 2:** Top 25 keywords with the strongest citation bursts.

Keywords	Strength	Starting Year	Ending Year	2010–2025
Charcoal	13.26	2010	2019	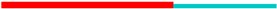
Black carbon	9.4	2010	2017	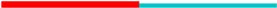
Physical property	3.19	2010	2016	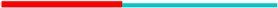
Dynamics	3.01	2011	2016	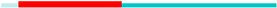
Soil	8.23	2012	2019	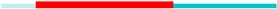
Pyrolysis temperature	6.03	2015	2019	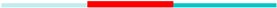
Slow pyrolysis	4.37	2015	2018	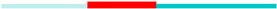
Yield	3.77	2015	2021	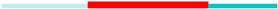
Polycyclic aromatic hydrocarbons	3.71	2015	2021	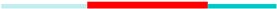
Amendment	3.56	2015	2018	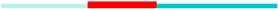
Temperature	3.74	2016	2019	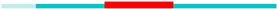
Malachite green	3.59	2016	2020	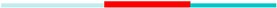
Productivity	3.16	2016	2017	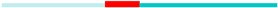
Retention	3.67	2017	2018	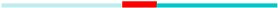
Greenhouse gas emissions	4	2018	2021	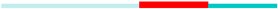
Conversion	3.06	2018	2019	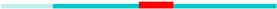
Temperature sensitivity	3.02	2018	2019	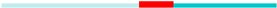
Heavy metals	4.53	2019	2020	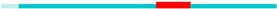
Enzyme activity	3.9	2019	2021	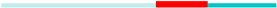
Drinking water	3.2	2020	2021	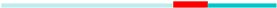
Low cost	4.09	2021	2022	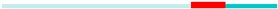
Contaminants	3.4	2021	2022	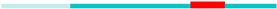
Zero-valent iron	3.89	2022	2023	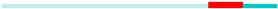
Cr(VI)	3.56	2023	2025	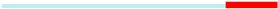
Carbonization	3.12	2023	2025	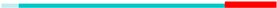

**Table 3:** Comparison of common RSM experimental design methods.

Design Method	Factor Levels	Number of Experiments	Effects Under Study	Scope of Application	Advantages	Disadvantages
Central Composite Design	5	$2^{k}+2k+n_{c}$	Main effects, interaction effects, and second-order effects	Optimization of 2–5 factors	High accuracy, rotational symmetry, and estimation of quadratic effects	Requires a large number of experiments, including tests under extreme conditions
Box–Behnken Design	3	$2k(k-1)+n_{c}$	Main effects, interaction effects, and second-order effects	Optimization of 3–5 factors	Fewer experiments; no experiments under extreme conditions	No axial points; limited to three levels

**Table 4:** Application of RSM in the optimization of biochar adsorption performance.

Pollutant Type	Pollutant	RSM	Optimization Variables	Model Fit Metrics	Optimal Conditions	Optimization Results	Consistency Between Predicted and Experimental Values	Number of Experiments	References
Heavy metals	Pb(II)	CCD	Solution pH, adsorbent dosage, humic acid (HA) concentration	R^2^ = 0.9923, model F = 299.29, *p* < 0.0001; outlier term *p* < 0.0001	pH = 8.65, adsorbent dosage 0.019 g, HA concentration 11.85 mg/L	CS-700 maximum adsorption capacity: 93.29 mg/g (Langmuir fit)	High agreement between predicted and measured values	31	[22]
	Cd(II)	CCD	Initial concentration, dosage, pH, adsorption time	R^2^ = 0.9951, adj-R^2^ = 0.9906, pred-R^2^ = 0.9749, F = 219.75	Concentration: 50 mg/L, dosage: 5 g/L, pH = 8, time 24 h	Removal efficiency: 93.4%, maximum adsorption capacity: 21.82 mg/g	Predicted: 92.97% vs. Experimental: 93.40%	30	[23]
	Hg(II)	BBD	Initial concentration, dosage, pH	R^2^ = 0.988, F = 56.652	Concentration: 14.3 mg/L, dosage: 4.5 g/L, pH = 3.9	Removal efficiency: 94.91% (predicted), 92.85% (experimental); maximum adsorption capacity: 85.8 mg/g	No significant difference between predicted and experimental values	17	[24]
	Cr(VI)	BBD	Solution pH, initial Cr(VI) concentration, adsorbent dosage, temperature, contact time	R^2^ = 0.9837, adj-R^2^ = 0.9837, pred-R^2^ = 0.9631, model F = 136.55, *p* < 0.0001; lack-of-fit *p* = 0.1223 (not significant)	pH = 2, initial concentration 60 mg/L, nZVI-biochar dosage 1.25 g/L, temperature 30 °C, contact time 60 min	Maximum removal efficiency: 80.22%; adsorption follows pseudo-second-order kinetics, fitted well with Temkin and Sips isotherms	High agreement, predicted values match well with experimental data, residuals follow normal distribution	46	[25]
Organic compounds	Phenol	BBD	pH, dosage, contact time	R^2^ = 0.97, adj-R^2^ = 0.90, F = 15.55, *p* = 0.004	pH = 6, dosage 0.1 g, contact time 20 h	Predicted adsorption capacity: 16.62 mg/g; experimental value: 17.38 mg/g	Deviation between predicted and experimental values < 5%	15	[26]
	Levofloxacin	BBD	Concentration, time, dosage, pH	adj-R^2^ = 0.9894, pred-R^2^ = 0.9880, F = 290.59	pH = 6, time = 90 min, dosage = 0.02 g	Maximum adsorption capacity: 1119.19 mg/g	The model exhibits high prediction accuracy, with all experimental data points falling within the 95% prediction interval	24	[27]
	Atenolol, acetaminophen, ketorolac, tetracycline	CCD	Solid-to-liquid ratio, pH, ionic strength	pred-R^2^ > 0.8; lack-of-fit test not significant	Atenolol: pH 6.5, solid-to-liquid ratio 0.34; Acetaminophen: pH 7.5; Ketorolac: pH 6.0; Tetracycline: pH 4.4	Atenolol 34.1%, acetaminophen 51.3%, ketorolac 55.9%, tetracycline 38.2%	All experimental values fall within the 95% prediction interval	18	[28]
	Methyl Orange (MO), Methylene Blue (MB) (binary system)	BBD	Contact time, adsorbent dosage, solution pH	MO: R^2^ = 0.9987, adj-R^2^ = 0.9968, pred-R^2^ = 0.9382, F = 527.56, *p* < 0.0001; MB: R^2^ = 0.9968, adj-R^2^ = 0.9921, pred-R^2^ = 0.8585, F = 215.62, *p* < 0.0001; lack-of-fit not significant	Contact time 12.89 min, adsorbent dosage 0.01 g, pH = 11, initial dye concentration 10 mg/L for each	MO experimental adsorption capacity: 13.86 mg/g; MB experimental adsorption capacity: 14.60 mg/g	Good agreement, relative deviation of MO ~5.9%, MB ~10.9%, confirmation test verifies model reliability	18	[29]
Non-metallic inorganic compounds	Phosphate	BBD	Pyrolysis time, pyrolysis temperature, FeCl_3_ concentration	F = 46.64, *p* < 0.0001; lack-of-fit *p* = 0.0629 (not significant); difference between adjusted R^2^ and predicted R^2^ < 0.2; precision = 24.29	Pyrolysis time 1.0 h, pyrolysis temperature 300 °C, Fe concentration 0.25 M	Optimized predicted adsorption capacity: 30.4 mg/g; maximum adsorption capacity from Langmuir fit: 31.80 mg/g	The predicted values agree well with the measured values	17	[30]
	Nitrate	CCD	GMBC ratio, pH	adj-R^2^ = 0.99 (5 days), pred-R^2^ = 0.99; adj-R^2^ = 0.99 (10 days), pred-R^2^ = 0.97; adj-R^2^ = 0.97 (20 days), pred-R^2^ = 0.96	pH = 4.0, GMBC concentration 3%	85.03% removed after 5 days, 76.63% after 10 days, 9.61% after 20 days	The predicted values agree well with the experimental values	13	[31]
	Ammonium nitrogen	BBD	Static: pH, adsorption time, adsorbent dosage. Dynamic: column height, feed flow rate, initial concentration	Static model F = 163.10, *p* < 0.0001, predicted R^2^ = 0.961; dynamic model F = 101.75, *p* < 0.0001, predicted R^2^ = 0.924; the misfit terms for both groups *p* > 0.05, not statistically significant	Static: pH = 7, adsorption time 310 min, adsorbent dosage 10 g/L; Dynamic: column height 10.0 cm, flow rate 1.04 mL/min, initial concentration 140.80 mg/L	Maximum removal efficiency under static conditions: 95.07%; maximum adsorption capacity from Langmuir fitting: 26.80 mg/g; maximum removal efficiency under dynamic conditions: 78.02%	The predicted values agree well with the experimental values	17	[32]
	Sulfate	BBD	Solution pH, initial sulfate concentration, adsorbent dosage	RCS: R^2^ = 0.9998, adj-R^2^ = 0.9988, pred-R^2^ = 0.9995, F = 3473.12, *p* < 0.05; CSB: R^2^ = 0.9992, adj-R^2^ = 0.9953, pred-R^2^ = 0.9981, F = 940.01, *p* < 0.05; Lack-of-fit not significant	RCS: pH = 7, initial concentration 150 mg/L, dosage 0.5 g; CSB: pH = 9.8, initial concentration 100 mg/L, dosage 0.1 g	RCS removal efficiency: 81.1%, Langmuir maximum adsorption capacity 61.35 mg/g; CSB removal efficiency: 89.5%, Langmuir maximum adsorption capacity 153.85 mg/g	High consistency, predicted values are in good agreement with experimental results, residuals show normal distribution	17	[33]

**Table 5:** Application of RSM in the optimization of biochar production processes.

Biomass Feedstock	RSM	Optimization Variables	Model Fit Metrics	Optimal Conditions	Optimization Results	Consistency Between Predicted and Experimental Values	Number of Experiments	References
Corn cobs	BBD	Pyrolysis temperature (300–600 °C), heating rate (5–15 °C/min), holding time (30–90 min)	R^2^ = 0.9949, Adj R^2^ = 0.9920; F = 349.08; *p* < 0.0001; MAPE = 0.85%	Pyrolysis temperature: 300 °C, heating rate: 5 °C/min, holding time: 30 min	Maximum biochar yield: 33.42%	Validation experiment MAPE = 1.89% (<5%), with high agreement between predicted and measured values	18	[50]
Rice husks	CCD	Pyrolysis temperature (298.87–551.13 °C), pyrolysis time (34.89–60.11 min), heating rate (2.98–8.02 °C/min)	R^2^ = 0.99, Adj R^2^ = 0.98 (yield model); F = 116.69; *p* < 0.0001	Pyrolysis temperature 432 °C, pyrolysis time 40 min, heating rate 4 °C/min	Biochar yield: 54.65%, HHV: 25.08 MJ/kg, energy density: 1.46, energy yield: 79.66%, fuel ratio: 2.10	The experimental values (yield 54.35%, HHV 24.66 MJ/kg, energy density 1.44, energy yield 78.25%) are close to the predicted values	20	[51]
Wood chips	BBD	Heating rate (5–15 °C/min), pyrolysis temperature (350–550 °C), residence time (0–60 min)	R^2^_(pred) = 0.9721, R^2^_(adj) = 0.9218; F = 19.2; *p* = 0.00231	Heating rate 12 °C/min, pyrolysis temperature 523 °C, residence time 49 min	Maximum phosphate removal rate: 93%	R^2^_(pred) = 97%, R^2^_(adj) = 92%; the test for nonlinearity was not significant (*p* = 0.149); the model is applicable	15	[52]
Chicken manure (fixed-bed reactor)	CCD	Pyrolysis temperature (200–500 °C), heating rate (5–20 °C/min), pyrolysis time (30–120 min)	R^2^ = 0.8815, Adj R^2^ = 0.7749; F = 8.27; *p* = 0.0014	Pyrolysis temperature 440 °C, heating rate 7 °C/min, pyrolysis time 115 min	Maximum BET specific surface area: 4.61 m^2^/g (experimental value); CO_2_ adsorption: 27.52 mg/g (raw charcoal), 66.62 mg/g (KOH-activated), 51.26 mg/g (HCl-activated); MB removal efficiency: 80%	Predicted specific surface area: 7.61 m^2^/g; experimental value: 4.61 m^2^/g; deviation: approximately 39%	20	[53]
Chicken manure (fluidized-bed reactor)	CCD	Reaction temperature (200–500 °C), heating rate (5–20 °C/min), reaction time (30–120 min)	R^2^ = 0.8717, Adj R^2^ = 0.7563; F = 7.55; *p* = 0.0020	Reaction temperature 400 °C, heating rate 5 °C/min, reaction time 110 min	Maximum BET specific surface area: 4.38 m^2^/g (experimental value); CO_2_ adsorption: 48.7 mg/g (raw carbon), 76.8 mg/g (HCl-activated), 85.7 mg/g (KOH-activated)	Predicted specific surface area: 5.30 m^2^/g; experimental value: 4.38 m^2^/g; deviation of approximately 17%	20	[54]
Cassava peel/sheep manure (co-pyrolysis)	CCD	Pyrolysis temperature (237.87–662.13 °C), residence time (26.89–48.10 min)	R^2^ = 0.9863 (50/50), 0.9924 (SM), 0.9797 (CP); F = 100.82, 183.66, 67.65; *p* < 0.0001	Pyrolysis temperature 237.87 °C, residence time 37.5 min	Maximum biochar yield: 93.2% (CP/SM 50/50); SM yield: 93.0%, CP yield: 88.0%	R^2^ > 0.97; no significant nonlinearity test (*p* > 0.05); COV 2.06–4.30%; the model explains > 97% of the variation	13	[55]
Digested sludge	CCD	Heating rate (12–14 °C/min), pyrolysis temperature (400–600 °C), heating time (20–240 min)	Adj R^2^ = 0.96; F-value significant (*p* < 0.0001); Adeq Precision = 20.87; C.V.% = 3.00%	Heating rate 13.23 °C/min, pyrolysis temperature 516 °C, heating time 192 min	Methane yield increased by 48% (cumulative methane yield: 250.71 mL/g VSS)	Predicted value: 256.73 mL/g VSS; experimental value: 250.71 mL/g VSS; high agreement	20	[56]
Slaughterhouse sludge + rice husks	BBD	Pyrolysis temperature (350–450 °C), heating rate (5–10 °C/min), holding time (1.5–2.5 h)	R^2^ = 0.9841, Adj. R^2^ = 0.9636; F = 48.01; *p* < 0.0001	Pyrolysis temperature 382 °C, heating rate 5 °C/min, holding time 2 h	Maximum U(VI) adsorption capacity: 159.27 mg/g; U(VI) removal efficiency: 83.04%	Predicted removal rate: 82.2%; experimental value: 83.04%; deviation < 1%; high degree of agreement	17	[57]
Municipal sludge	CCD	Pyrolysis temperature (450–650 °C), heating rate (5–50 °C/min), residence time (30–180 min)	Yield: Adj R^2^ = 0.9506, Pred R^2^ = 0.8369, *p* < 0.0001; CO_2_ adsorption: Adj R^2^ = 0.7396, Pred R^2^ = 0.5892, *p* < 0.0001 (2FI model)	Pyrolysis temperature: 490.54 °C, heating rate: 14.12 °C/min, residence time: 149.6 min (priority CO_2_ adsorption)	Maximum biochar yield: 47.8 wt%; maximum CO_2_ adsorption capacity: 0.514 mol CO_2_/kg; optimal biochar specific surface area: 124 m^2^/g	TGA experiments: average relative error in yield ~0.7%, CO_2_ adsorption error ~4%; significant deviations observed in scale-up experiments (yield 6%, CO_2_ adsorption 33%)	20	[58]

**Table 6:** Application of RSM in the optimization of biochar modification processes.

Modification Method	Biomass Feedstock	RSM	Optimization Variables	Model Fit Metrics	Optimal Conditions	Optimization Results	Consistency Between Predicted and Experimental Values	Number of Experiments	References
KOH	Pulp sludge	BBD	Pyrolysis temperature (700–900 °C), holding time (1–3 h), sludge: KOH ratio (1:1–1:2)	R^2^ = 92% (SSA), 80% (Smicro), 97% (Smeso)	718 °C, 3 h, biomass: KOH = 1:1 (comprehensive optimization)	Maximum SSA 885 m^2^/g, DCF adsorption 357 mg/g, AMX adsorption 305 mg/g	The model fit is reasonable, but there is a significant “lack of fit” for SSA and Smicro	15	[67]
KOH	Young coconut shell waste	CCD	Carbonization temperature (400–600 °C), carbonization time (90–200 min), KOH concentration (2–5 M)	Iodine value: adjusted R^2^ = 0.7361, F = 6.89, *p* = 0.0029; yield: adjusted R^2^ = 0.5207; MB adsorption: adjusted R^2^ = 0.4764	400 °C, 165.84 min, 4.068 M KOH	Iodine value 1398.2 mg/g, MB adsorption efficiency 93.9%, yield 25.7%	Iodine value deviation: 2.48 mg/g; yield deviation: 1.88 wt%; MB adsorption deviation: 0.41% (deviation < 2%)	20	[68]
NaOH	Jute fiber	CCD	Pyrolysis temperature (300–600 °C), reaction time (60–180 min), NaOH impregnation ratio (1–3 *w*/*w*)	R^2^ = 0.956 (yield), 0.976 (Cd removal), 0.964 (adsorption capacity), 0.969 (SSA); F = 23.83 (yield), 45.3 (Cd removal), 30.08 (adsorption capacity), 34.78 (SSA)	550 °C, 180 min, 1:1 NaOH impregnation ratio	Yield 28.60%, Cd removal rate 69.82%, adsorption capacity 23.48 mg/g, SSA 160.44 m^2^/g	The experimental values agree well with the model predictions (agreement degree: 66.70%)	20	[69]
H_3_PO_4_	Phellinus linteus residue	BBD	H_3_PO_4_:IOR mass ratio (1–5), pyrolysis temperature (300–400 °C), pyrolysis time (0.5–5.5 h)	R^2^ = 0.9843, adjusted R^2^ = 0.9642, predicted R^2^ = 0.7921, F = 48.82, *p* < 0.0001, adequate precision = 19.61, C.V.% = 4.11	Mass ratio 3.91, 374 °C, 166 min	MB adsorption: 951.56 mg/g, SSA: 2014.51 m^2^/g, TC adsorption: 1515.46 mg/g	The experimental MB adsorption of 951.56 mg/g is close to the predicted value of 971.00 mg/g (a deviation of approximately 2%)	17	[70]
Al	Food waste	BBD	Pyrolysis temperature (300–600 °C), pyrolysis time (0.5–3.5 h), Al concentration (2–6%)	R^2^ = 0.904, F = 28.35, *p* < 0.0001, adjusted R^2^ = 0.872, predicted R^2^ = 0.776, adequate precision = 18.4	300 °C, 0.5 h, 6% Al concentration	Maximum phosphate adsorption capacity: 197.8 mg/g; phosphate removal rate: 99.6%	The predicted R^2^ (0.776) is consistent with the adjusted R^2^ (0.872), indicating a reliable model	17	[71]
H_2_SO_4_ + Fe	Nut shells	CCD	Activation: H_2_SO_4_ concentration (1–5 N), nut shell mass (10–20 g), activation time (30–60 min); loading: Fe^3+^ concentration (0.05–0.1 M), impregnation time (60–120 min)	Activation: MB value R^2^ = 93.99%^,^ iodine value R^2^ = 95.50%; loading: MB value R^2^ = 92.75%, iodine value R^2^ = 95.41%	Activation: 1 NH_2_SO_4_, 10 g walnut shells, activated for 30 min; loading: 0.075 M iron salt, 6 g Morinda officinalis, impregnated for 60 min	Specific surface area reached 361.70 m^2^/g, with a malachite green removal rate of 97.08%	Under optimal conditions, the deviation between experimental and predicted values was <5%, indicating good agreement with the two-stage model	20	[72]
CO_2_	Palm kernel shells	BBD	Activation temperature (750–950 °C), holding time (60–120 min), CO_2_ flow rate (150–450 mL/min)	Y_1_: F = 148.84, *p* < 0.0001; Y_2_: F = 24.14, *p* = 0.0002; lack of fit *p* > 0.05	Activation temperature 950 °C, holding time 60 min, CO_2_ flow rate 150 mL/min	Maximum yield: 61.37 wt.%, CO_2_ adsorption capacity: 2.493 mmol/g (superior to commercial activated carbon at 1.9 mmol/g)	Deviation between predicted and experimental values < 15%; the model is reliable	17	[73]
CO_2_	Used diapers	BBD	Activation temperature (700–900 °C), holding time (60–120 min), CO_2_ flow rate (150–250 mL/min)	R^2^ = 0.9972, R^2^adj = 0.9936, F = 275.56, *p* < 0.0001; lack of fit *p* = 0.4732	Activation temperature: 900 °C, holding time: 90 min, CO_2_ flow rate 150 mL/min	BET surface area: 1235.32 m^2^/g (compared to only 479.79 m^2^/g for biochar); pore diameter: 2.13 nm	R^2^ = 0.9972; the predicted values show extremely high agreement with the experimental values	17	[74]
Steam	Flaxseed cake	CCD	Activation temperature (600–900 °C), steam flow rate (5–15 mL/h), activation time (60–120 min)	R^2^ = 0.998, R^2^adj = 0.997, R^2^pred = 0.990, F = 865.05, *p* < 0.0001	Temperature 899 °C, time 75 min, steam flow rate 14 mL/h	BET surface area: 722 m^2^/g; PFOA removal efficiency: 88.9%	Predicted value: 738 m^2^/g; measured value: 722 m^2^/g; error: 2.2%	20	[75]
Bacillus cereus MZ-1	Corn stover	BBD	Pyrolysis temperature (300–700 °C), bacterial strain dosage (1–5 times), immobilization time (6–24 h)	R^2^ = 0.9844, R^2^adj = 0.9644, F = 49.14, *p* < 0.0001	BC500, 3.5-fold biochar solution of bacterial suspension (OD_600_ = 1), immobilization time 18 h	Maximum imazethapyr removal efficiency: 79.85% (compared to 52.65% for free-living strains)	Predicted value: 80.82%, measured value: 79.85%, deviation of approximately 1.2%, high agreement	17	[76]
Bacillus consortium	Oil palm kernel shells	CCD	Temperature (28–38 °C), stirring speed (70–200 rpm), pH (3–12), starch concentration (0–20% *w*/*w*)	R^2^ = 0.998, R^2^adj = 0.996, F = 531.45, *p* < 0.05	Temperature 33 °C, stirring speed 135 rpm, pH 7.5, starch 10% (*w*/*w*)	Maximum Bacillus population: 2.37 × 10^8^ CFU/mL; maintained at 10^7^ CFU/g after 4 months of storage	R^2^ = 0.998; the model predictions closely matched the experimental values	30	[77]

## Data Availability

The bibliometric record set and the data extracted from the studies summarized in Table 4, Table 5 and Table 6 are available from the corresponding author upon reasonable request. The literature search strategy, screening records, and bibliometric datasets used to generate Figure 1, Figure 2, Figure 3, Figure 4, Figure 5 and Figure 6 are documented in Section 2 and can be provided on request.
